# Unraveling Pathway Complexity in the Supramolecular Polymerization of Z‑Shaped Perylenediimides: From Kinetic *H*‑Aggregates to Thermodynamic Null Supramolecular Polymers

**DOI:** 10.1021/jacs.5c08436

**Published:** 2025-07-07

**Authors:** Alfonso J. Schwalb, Cristina Naranjo, Alberto Fernández-Alarcón, Fátima García, Enrique Ortí, Juan Aragó, Luis Sánchez

**Affiliations:** † Departamento de Química Orgánica, Facultad de Ciencias Químicas, 16734 Universidad Complutense de Madrid, 28040 Madrid, Spain; ‡ Instituto de Ciencia Molecular (ICMol), 16781 Universitat de València, 46980 Paterna, Spain

## Abstract

This work reports the synthesis of Z-shaped PDI (Z-PDI) **1** and explores its self-assembly behavior. The lateral trialkoxybenzamide moieties in compound **1** promote the formation of metastable monomeric units (M*) through intramolecular hydrogen bonds, which undergo kinetically controlled supramolecular polymerization. This process exhibits pathway complexity, yielding *H*-type aggregates (*AggI*_*H*_) under kinetic control and, remarkably, null aggregates (*AggII*_*n*_) under thermodynamic control. The conversion follows a competitive pathway, in which both aggregated states compete for the free monomeric species. A combination of experimental data and theoretical calculations reveals that the formation of *AggI*_*H*_ is governed by the intermolecular hydrogen bonding between amide groups and the π-stacking of the aromatic cores. The thermodynamically favored null aggregate *AggII*_*n*_ also arises from the same noncovalent interactions but its unique nature stems from a balance between Coulombic and charge-transfer interactions—similar in magnitude yet opposite in sign, resulting in an optical absorption profile nearly identical to that of the monomer. The living supramolecular polymerization of Z-shaped PDI **1** enables the transition from kinetically trapped to thermodynamically stable aggregates. These findings highlight the critical role of the molecular design in achieving null aggregation and pathway complexity, while emphasizing the importance of π-overlap, intermolecular distance, and chromophore orientation in determining the nature of the resulting supramolecular assemblies.

## Graphical abstract

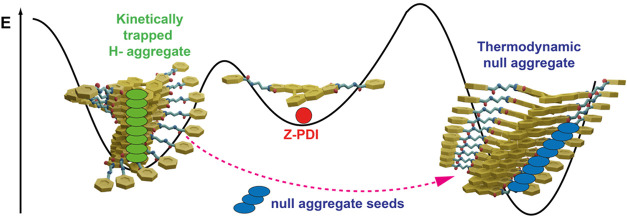

## Introduction

The self-assembly of amphiphilic porphyrins reported by Aida et al. in 1988 ^1^ and the complementary interaction of a ditopic uracil and a ditopic 2,6-diacylamino-pyridine reported by Lehn and co-workers in 1990 ^2^ opened the field of supramolecular polymers, harnessing the knowledge provided by the more ample areas of supramolecular chemistry and self-assembly.^3^ In addition, the advent of mathematical mass-balance models valid to investigate the thermodynamics of the supramolecular polymerization mechanisms^4^ and the development of kinetically (KC) and thermodynamically controlled (TC) examples of supramolecular polymerizations have provided a remarkable boost to the field.^5^ Since the seminal work reported by Aida and co-workers,^1^ a large number of supramolecular polymers has been built up by using a variety π-conjugated moieties such as porphyrins,^1,6^ oligomers,^7^ squaramides,^8^ metal complexes,^9^ 4,4-difluoro-4-bora-3a,4a-diaza-*s*-indacene (BODIPY) dyes,^10^ rylenes,^11^ and, very especially, perylenediimides (PDIs).^12^ The optical response of these scaffolds upon aggregation, which strongly conditions the final functionality of the aggregated species, differs from that of the monomeric units and is related to the electronic communication between the chromophores. The electronic communication in the aggregate is conditioned by the π-overlap, the intermolecular distance, and the relative spatial orientation of the chromophores, and allows to define different aggregated species, often grouped in *H*- or *J*-type aggregates following Kasha’s classification, in which the intermolecular interaction of the monomeric units is defined by the Coulombic coupling (*J*_Coul_). This coupling arises from the long-range electrostatic-like interactions, an effect that can persist up to hundreds of nanometers.^13,14^ The interaction of transition dipoles in aggregated species provokes exciton splitting and the concomitant spectral shifts or splitting of the absorption bands.

The Kasha’s exciton theory identifies two opposite situations for aggregated species: (i) the *J*-type aggregates with a head-to-tail arrangement of the monomeric species, in which a red-shifted absorption maximum and an enhanced radiative decay rate are observed due to the transition to a lower energy state, and (ii) the cofacial *H*-type aggregates, in which a blue-shifted absorption maximum relative to the monomers with suppressed fluorescence emission is observed since the transition to a higher energy state takes place. The sign of *J*_Coul_ dictates the formation of conventional *H* and *J* aggregates.^15,16^ Interestingly, between these two opposite arrangements, Kasha also predicted two situations in which the aggregated species presented a null Coulombic coupling. The first one corresponds to parallelly arranged transition dipoles with a slip angle (α) of 54.71°, and the second one is ascribed to orthogonally cross-stacked transition dipoles yielding minimal exciton interactions.^14a,16b^ The long-range Coulombic coupling model defined by Kasha has been recently complemented by the incorporation of short-range, charge-transfer (CT)-mediated interactions, initially described by Harcourt, Scholes, and Ghiggino,^17a^ and further extended by Hestand and Spano by including the effect of vibrations on the optical properties of the aggregated species.^17b^ The CT-mediated (*J*_CT_) coupling stems from the overlap between the HOMOs and LUMOs of the stacked chromophores, and is highly sensitive to the slip angle of the monomeric units in the aggregate.^17b^ In short, classification of aggregated species into *H*- or *J*-type is too simplistic, and the combined effect of short-range CT and long-range Coulombic couplings should be considered.^16b^ Although still complicated, *null aggregates* may emerge, in this context, as a consequence of destructive interference between long- and short-range interactions and an appropriate energy offset between the relevant low-lying excited states. These aggregates are characterized by exhibiting spectral properties reminiscent of those shown by the monomeric species.

Herein, we report on a highly emissive Z-shaped PDI (Z-PDI **1** in Figure 1a) endowed with lateral trialkoxybenzamide units that favors the formation of metastable monomeric units (M*) and, consequently, the kinetic control of the supramolecular polymerization of this emissive scaffold. The supramolecular polymerization of Z-PDI **1** relies on intermolecular H-bonding interactions between the amide functional groups that are reinforced by the π-stacking of the central aromatic moiety. The supramolecular polymerization of Z-PDI **1** is strongly conditioned by the solvent. Thus, in pristine methylcyclohexane (MCH), the absorption pattern of the aggregated species presents a fine structure that matches the absorption pattern registered for the free monomeric species in chloroform solution but is slightly red-shifted (Figure 1b). This absorption pattern could be diagnostic of the unprecedented formation of a *null aggregate*.^16b,18^ However, in a mixture of MCH and toluene (Tol), the M* species yields a kinetically controlled supramolecular polymerization in which two different aggregated species, *AggI*_*H*_ and *AggII*_*n*_, are detected. As occurs in pristine MCH, the absorption vibronic pattern of the thermodynamically controlled supramolecular polymer *AggII*_*n*_ coincides in shape with that of the monomeric species (Figure 1b). In contrast, the kinetically controlled *AggI*_*H*_ presents a clear hypso- and hypochromic shift in the absorption pattern diagnostic of a *H*-type aggregate (Figure 1b). Theoretical calculations contribute to clarify the noncovalent interactions operating in the formation of both aggregated species and the contribution of Coulombic and CT couplings. Interestingly, the thermodynamically controlled *AggII*_*n*_ aggregate shows a slipped arrangement of the monomeric units (Figure 1c), in which both Coulombic and CT couplings are relevant. In contrast, the Coulombic coupling is predominant in the kinetically controlled *AggI*_*H*_ aggregate, in which the adjacent monomeric units are rotated giving rise to a columnar aggregate (Figure 1d). Therefore, the pathway complexity exhibited by Z-PDI **1** strongly biases the balance between short- and long-range excitonic interactions. The kinetics of the *AggI*_*H*_ → *AggII*_*n*_ conversion is found to be very slow, since more than 24 h is required to achieve a complete transformation. However, the application of mechanical stirring accelerates the conversion, being thus completed within 6 h. The kinetic studies demonstrate that the *AggI*_*H*_ → *AggII*_*n*_ conversion follows a competitive pathway. A seeded-induced living supramolecular polymerization (LSP) was also performed to transform *AggI*_*H*_ into *AggII*_*n*_ by adding seeds of the latter to a solution of the former.^19^ The studies presented herein highlight the sensitivity of Coulombic and CT interactions to subtle variations of the relative spatial orientations of the chromophores in the aggregate. The pathway complexity shown by Z-PDI **1** allows the study of different aggregates, including the rarely, experimentally described *null aggregate*, contributing to deepening the understanding of the effects that Coulombic and CT interactions have on the electronic and photophysical properties of supramolecular polymers.

**Figure 1:**
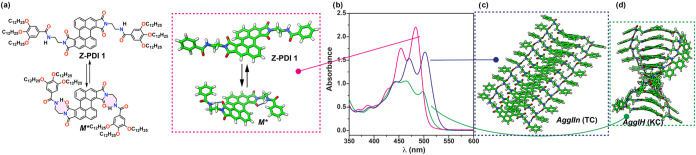
(a) Chemical structure and optimized geometries of Z-PDI **1** in different conformations (the terminal alkoxy chains have been omitted for clarity). (b) UV–vis spectra of **1** in MCH/Tol 1/1, *c_T_* = 100 μM, showing the absorption pattern of the monomeric species (pink), the kinetically controlled aggregated species *AggI*_*H*_ (green), and the thermodynamically controlled aggregated species *AggII*_*n*_ (blue). Optimized geometry of a decamer model of *AggII*_*n*_ (c), and a decamer model of *AggI*_*H*_ (d).

## Results and Discussion

### Synthesis and Self-Assembly in Pristine Methylcyclohexane

Z-PDI **1** was synthesized starting from commercially available 1,5-diamino-9,10-anthraquinone in a multistep protocol (Scheme S1). The synthesis of target **1** requires the preparation of dianhydride **6** that first involves a Sandmeyer reaction to transform 1,5-diamino-9,10-anthraquinone into 1,5-diiodo-9,10-anthraquinone (**2**).^20^ Following a modified strategy, we performed a Suzuki coupling between **2** and (*E*)-styrylboronic acid to prepare previously reported anthraquinone **3** in good yield. Distyrylanthraquinone **3** underwent reductive ozonolysis to afford 9,10-anthraquinone-1,5-dicarbaldehyde (**4**) which was subjected to a Wittig-Knoevenagel benzannulation to yield tetraester **5**.^20^ The acidic hydrolysis of **5** gave rise to dianhydride **6** that, upon reaction with benzamide **7** ^21^ in the presence of Zn­(AcO)_2_ and imidazole, yielded the final Z-PDI **1**. The newly reported compounds have been fully characterized by utilizing standard techniques (Supporting Information).

To investigate the supramolecular polymerization of Z-PDI **1**, and taking into account the well-known trend to form seven-membered pseudocycles in related PDIs where the amide functional groups give rise to intramolecular H-bonds, the FTIR spectrum of **1** was first registered in CHCl_3_, a good solvent that favors the solvation of the monomeric units.^22^ The spectrum shows two bands for the NH stretching at 3449 and 3411 cm^–1^, ascribable to the free and intramolecularly H-bonded amides, respectively (Figure 2a).^23^ Additional evidence of the formation of the seven-membered pseudocycles (structure M* in Figure 1a) was obtained from the ^1^H NMR spectra of a diluted CDCl_3_ solution of **1** (*c_T_* = 1 mM) at different temperatures (Figure S1). Upon heating, the spectra show the upfield shift of the triplet ascribable to the amide protons, due to the rupture of the intramolecular H-bonds formed between the NH of the peripheral benzamide units and one of the carbonyls of the imide groups. Unlike the resonances ascribable to the amides, the resonances of all of the aliphatic and most of the aromatic protons do not experience any change upon increasing the temperature, since in these experimental conditions, the system is molecularly dissolved. Interestingly, the doublet corresponding to the protons at the bay position of the perylene core shows a deshielding effect that could be accounted for by considering the close proximity of the peripheral trialkoxybenzamide units to the central Z-PDI core upon formation of the seven-membered pseudocycles (Figure S1). This hypothesis is confirmed by a ROESY experiment registered in CDCl_3_ at *c_T_* = 1 mM (Figure 2b). Despite the high dilution, the ROESY 2D spectrum reveals through-space intramolecular contacts, like those observed between the methylenes of the trialkoxybenzamides and the aromatic resonances or between the singlet ascribable to the aromatic ring of the benzamides and the doublet of the proton at the bay position, that can be accounted for only by considering the folding of the peripheral groups toward the central perylene moiety.

**Figure 2:**
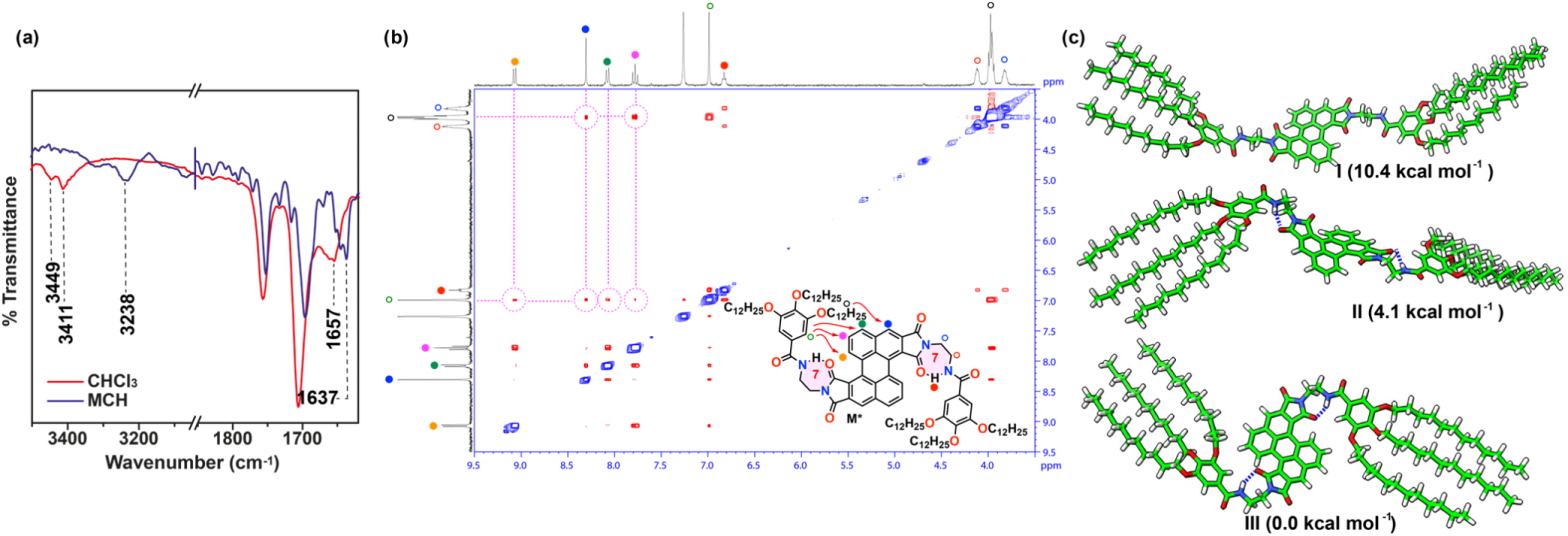
(a) Partial solution FTIR spectra of Z-PDI **1** showing the stretching NH and Amide I bands in CHCl_3_ and MCH at *c_T_* = 1 mM. (b) ROESY NMR spectrum (CDCl_3_, 300 MHz, 1 mM, 293 K) of **1**. The dotted lines highlight intramolecular through-space coupling signals. (c) GFN2-xTB-optimized geometries calculated for the three conformers (I, II, and III) of the Z-PDI **1** monomer, depicting the relative energy of each conformer.

To further study the geometric characteristics of **1**, the minimum-energy structures of this Z-PDI in three different conformations were calculated theoretically using the semiempirical GFN2-xTB method.^24^ The first conformation corresponds to an extended structure in which intramolecular H-bonding interactions are not present (Figure 1a top and conformer I in Figure 2c). The other two conformations correspond to the intramolecularly H-bonded monomeric species in which the peripheral benzamide units are pointing outward or inward (conformers II and III in Figure 2c, respectively). Theoretical calculations reveal that the formation of the seven-membered pseudocycles stabilize the structure and conformers II and III are more stable than the extended conformer I, conformer III being the most stable. These outcomes are in line with the experimental evidence inferred from variable-temperature (VT) ^1^H NMR and ROESY spectra (Figures S1 and 2b) suggesting that conformer III, with the peripheral benzamide units pointing inward, is more stable than conformer II. It is worth mentioning that, in good agreement with that observed for referable Z-PDIs,^25^ the central core of Z-PDI **1** is distorted from planarity with the planes defined by the two PDI naphthalene moieties forming an angle θ of around 10–12° (Figure S2). This distortion from planarity gives rise to the formation of two atropisomers. Nevertheless, the inversion barrier between these isomers is predicted to be small (3.85 kcal mol^–1^), which implies the spontaneous flipping of both configurations at room temperature (Figure S2).

After studying the structural characteristics of the monomeric species of Z-PDI **1**, we investigated its self-assembling features. First, we registered the ^1^H NMR spectra of **1** in CDCl_3_ at different concentrations. As occurs in referable PDI-based supramolecular polymers,^21,23a,26^ all the aromatic resonances shield upon increasing the concentration. In contrast, the triplet corresponding to the amide functional groups shifts downfield upon increasing the concentration (Figure S3). These opposite shifts observed in the ^1^H NMR spectra can be justified by considering the π-stacking of the aromatic central moieties and the operation of an array of intermolecular H-bonding interactions. The stretching NH and Amide I bands of **1** in a poor solvent like MCH, observed in the FTIR spectrum centered at 3238 and 1637 cm^–1^, respectively, confirm the formation of intermolecular H-bonding interactions (Figure 2a).^23,26^

To further elucidate the supramolecular polymerization of Z-PDI **1**, we registered the variable-temperature (VT) UV–vis absorption spectra in MCH solution (*c_T_* = 10 μM). To our surprise, the absorption pattern of Z-PDI **1** in MCH at 20 °C is analogous but slightly batochromically shifted to that observed in MCH at 90 °C (Figure 3a) and that registered for **1** in a good solvent like CHCl_3_ (Figure S4), both corresponding to the molecularly dissolved species. This similar absorption pattern contrasts with that previously reported for PDI-based supramolecular polymers, for which the aggregated species formed upon self-assembly shows a clear broadening of the absorption pattern compared to the monomeric species,^12b,27^ and could be indicative of the lack of supramolecular polymerization in MCH at 20 °C. However, atomic force microscopy (AFM) imaging of a 10 μM MCH solution of Z-PDI **1**, spin-coated onto a highly oriented pyrolytic graphite (HOPG) surface, shows a very dense network of fibrillar structures of around 4 nm height (Figure 3b,c and S5). These images, summed with the variable concentration NMR and the FTIR experiments, constitute an unambiguous proof of the formation of supramolecular polymers in these experimental conditions.

**Figure 3:**
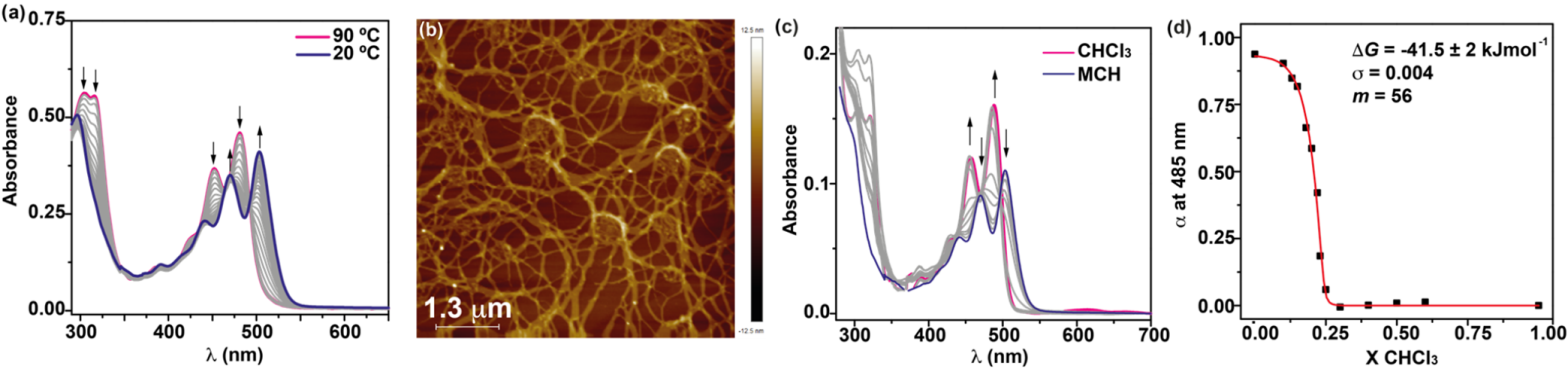
(a) UV–vis spectra of Z-PDI **1** in MCH at different temperatures (*c_T_* = 40 μM). (b) Height AFM image of the fibrillar aggregates formed by **1** in MCH onto HOPG as a surface (*c*_*T*_ = 10 μM). (c) UV–vis spectra of Z-PDI **1** in MCH and CHCl_3_ mixtures at different ratio (*c*_*T*_ = 10 μM). (d) Degree of aggregation extracted from (c) versus the molar fraction of CHCl_3_ for Z-PDI **1**. The black arrows in panels (a, c) indicate the changes in the UV–vis spectra upon cooling the solution (a) or upon increasing the amount of CHCl_3_ (c). The red line in panel (d) corresponds to the fitting to the solvent denaturation (SD) model.

Despite the similarity of the UV–vis spectra of **1** in MCH at different temperatures, several crossing points at ∼500, 475, and 350 nm are observed, diagnostic of the presence of different monomeric and aggregated species (Figure 3a). In fact, plotting the variation of the absorbance at 504 nm versus temperature gives rise to a nonsigmoidal curve (Figure S6a) that cannot be accurately fitted to the one-component equilibrium (EQ) model,^28^ most probably due to the presence of the above-mentioned hydrogen-bonded metastable monomeric species M* that affords a kinetically controlled supramolecular polymerization (see next section). Further confirmation of the influence of the formation of the M* species is inferred from the clear hysteresis recorded for the heating and cooling curves in MCH, from which the temperature of elongation *T*_e_ (i.e., the temperature at which the nucleation regime changes to the elongation) changes significantly (Figure S6b,c).^29,30^ Taking into account this kinetic control, some thermodynamic parameters were derived by performing a solvent denaturation (SD) experiment, in which the UV–vis spectra of mixtures of solutions of Z-PDI **1** at different ratios of the good solvent CHCl_3_ and the poor solvent MCH, while keeping constant the total concentration, were registered (Figure 3d). Plotting the degree of aggregation α versus the molar fraction of CHCl_3_ yields a nonsigmoidal curve that can be fitted to the SD model reported by Meijer and co-workers in 2012.^22^ The application of the SD model provides values for the Gibbs free energy of Δ*G* = −41.5 kJ mol^–1^, a degree of cooperativity σ = 0.004, and a factor *m*, that indicates the influence of the good solvent on the stability of the supramolecular polymers, of 56 (Figure 3d).

### Pathway Complexity Yielding *H*-Type and Null Aggregates

The formation of metastable monomers is often related to the operation of pathway complexity, from which a unique monomeric species leads to different aggregated states.^23b,26a,26c,31^ It is also well-stablished that the experimental conditions, and very especially the solvent,^21,26a^ can exert a crucial role in the differentiation process during the supramolecular polymerization. Thus, to further investigate the supramolecular polymerization of Z-PDI **1**, we modified the experimental conditions for supramolecular polymerization. Instead of using pristine MCH, we prepared a solution of **1** in MCH/Tol 1/1 at *c*_*T*_ = 100 μM and registered the corresponding UV–vis spectrum at 20 °C. At these experimental conditions, the optical absorption pattern coincides with that observed for the aggregated species formed in MCH at 20 °C with maxima at 504 and 470 nm (Figure 3a). Heating this solution to 90 °C generates an absorption pattern with maxima at λ = 484 and 455 nm, coincident with that observed for the monomeric species (pink line in Figure 4a). Interestingly, cooling this solution to 20 °C by applying a cooling rate of 1 °C min^–1^ affords a hypo- and hypsochromically shifted absorption pattern that is usually ascribed to the formation of *H*-type aggregates (green line in Figure 4a). Plotting the variation of the absorbance at λ = 484 nm versus temperature results in a nonsigmoidal curve that, once again, cannot be accurately fitted to the one-component EQ model (green squares in Figures 4b and S7b).^28^ In addition, heating this solution to 90 °C by applying the same heating rate of 1 °C min^–1^ affords a dissimilar curve in which two different transitions are observed (Figure S7b). All of these findings can be rationalized by the kinetic control exerted by the metastable M* monomeric units.

**Figure 4:**
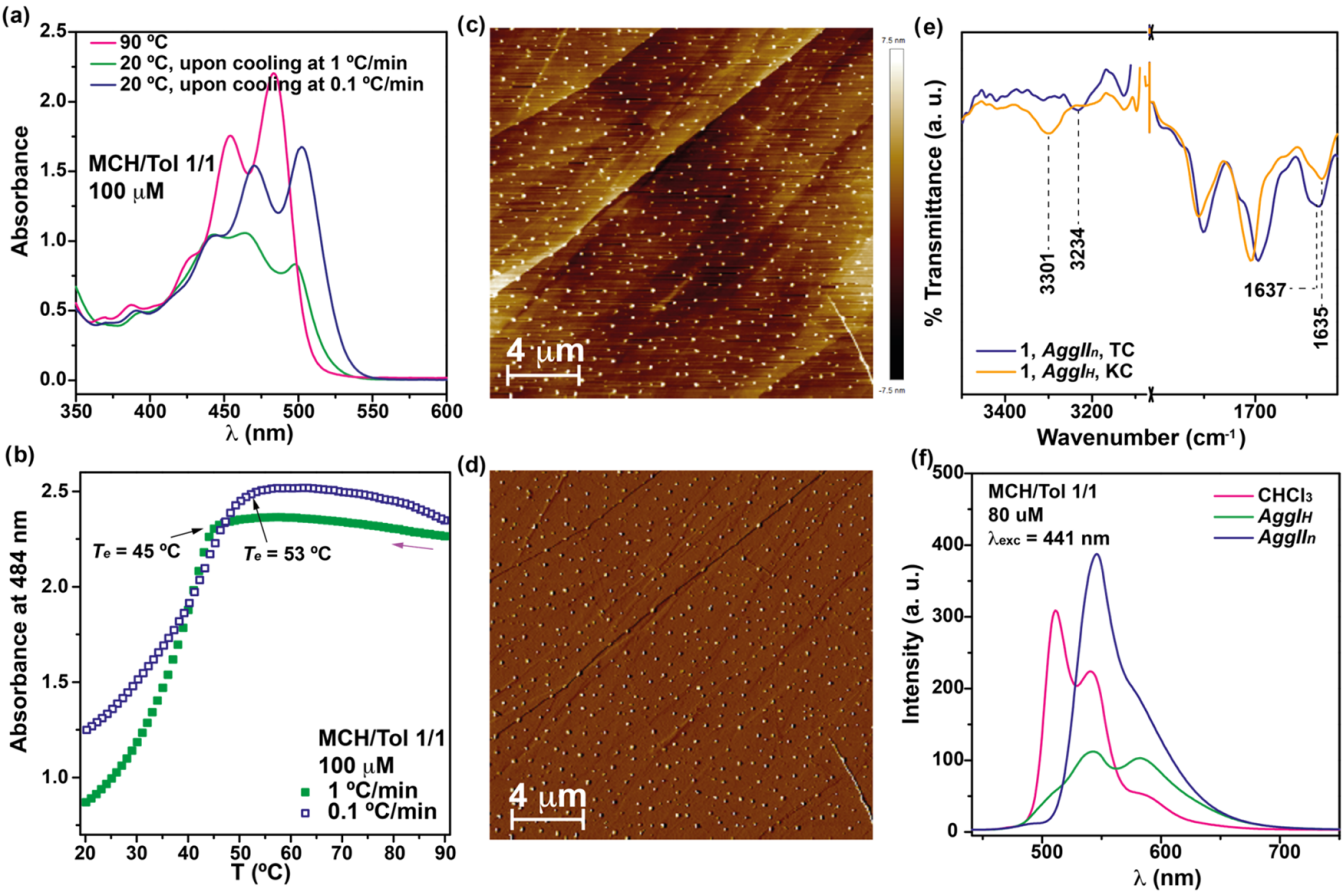
(a) Absorption pattern of the monomeric (pink line), *AggI*_*H*_ (green line), and *AggII*_*n*_ (blue line) species formed by Z-PDI **1** in MCH/Tol 1/1 (*c*_*T*_ = 100 μM). (b) Cooling curves of a 100 μM solution of **1** in MCH/Tol 1/1 upon applying a cooling cycle of 1 and 0.1 °C min^–1^, respectively. Height (c) and phase (d) AFM images of the nanoparticles formed by **1** in a MCH/Tol 1/1 solution (HOPG; *c*_*T*_ = 10 μM; 20 °C). (e) Partial FTIR spectra of *AggI*_*H*_ and *AggII*_*n*_ showing the stretching NH and Amide I bands in MCH/Tol 1/1 at *c*_*T*_ = 1 mM. (f) Emission spectra of the monomeric (pink line), *AggI*_*H*_ (green line), and *AggII*_*n*_ (blue line) species formed by Z-PDI **1** in MCH/Tol 1/1 (*c*_*T*_ = 80 μM, λ_exc_ = 441 nm).

To our delight, the application of a slower cooling rate (0.1 °C min^–1^), which allows achieving thermodynamic control in the supramolecular polymerization of **1**, yields an aggregated species with the same absorption pattern than that observed previously in MCH (blue line in Figure 4a). Once again, the cooling curve associated with this transformation displays a nonsigmoidal shape and it cannot be fitted to the one-component EQ model (blue squares in Figure 4b). As observed in MCH, the elongation temperature *T*_e_ at a 0.1 °C min^–1^ cooling rate (53 °C) is different to that recorded for the formation of the kinetically controlled aggregated species at 1 °C min^–1^ (45 °C, Figure 4b). Therefore, while the aggregated species formed in pristine MCH or by applying a cooling rate of 0.1 °C min^–1^ to a solution of **1** in MCH/Tol 1/1 is assigned to the thermodynamically controlled aggregate species (*AggII*_*n*_ in Figure 1), the absorption pattern obtained upon cooling a MCH/Tol 1/1 solution of **1** at 1 °C min^–1^ is ascribed to a kinetically controlled aggregated species (*AggI*_*H*_ in Figure 1). The morphology of the *AggI*_*H*_ aggregate has also been visualized by AFM. In this case, a large number of nanoparticles of 10 nm height are observed upon spin-coating a 10 μM solution of **1** in MCH/Tol 1/1 and applying a heating/cooling cycle at 1 °C min^–1^ (Figures 4c,d and S8). To further characterize the spectroscopic features of both aggregated species, the FTIR spectra of both the kinetically *AggI*_*H*_ and the thermodynamically controlled *AggII*_*n*_ aggregated species in the 1/1 MCH/Tol solution were registered (Figure 4e). The FTIR spectra show the NH stretching bands centered at 3301 and 3234 cm^–1^ for *AggI*_*H*_ and *AggII*_*n*_, respectively, and the Amide I bands around 1635 cm^–1^ for both *AggI*_*H*_ and *AggII*_*n*_. These wavenumber values, together with the changes observed in the absorption pattern, are diagnostic of the operation of intermolecular H-bonding interactions between the amide functional groups and the π-stacking of the aromatic cores in the formation of both the *AggI*_*H*_ and *AggII*_*n*_ species.

The bathochromic shift observed in the UV–vis spectra in the aggregated state in MCH and also in the thermodynamically controlled *AggII*_*n*_ in the MCH/Tol 1/1 mixture could be indicative of the formation of a *J-*type aggregate.^12b,13,14^ In fact, the emission spectrum of **1** in this mixture experiences a slight increase of the emission intensity in comparison to that registered for this Z-PDI in the monomeric state (Figure 4f). In fact, the derived fluorescence quantum yields for both the monomeric species and the *AggII*_*n*_ aggregate present almost identical values (Table S1 in the Supporting Information). On the contrary, the emission of the kinetically controlled *AggI*_*H*_ species is quenched compared to the monomeric species (Figure 4f). This aggregation caused quenching (ACQ) effect is associated with the formation of classical *H-*type aggregates and is corroborated by the lower fluorescence quantum yield measured for *AggI*_*H*_ (Table S1). Noteworthy, both the absorption and the emission patterns observed for the thermodynamically controlled *AggII*_*n*_ do not exactly match with a classical *J*-type aggregate and could be diagnostic of the formation of an unprecedented *null* aggregate.^14a,16b^ It is worth mentioning that, to the best of our knowledge, the majority of the few examples of null aggregates reported in the literature has been detected as crystalline samples^32^ except the ones reported by Würthner and co-workers, related to PDI-based folda-dimers and folda-tetramers,^33^ and those described by Cao, Xie, and Guldi on diketopyrrolopyrrole-based oligo-grids,^34^ in which the formation of the null-dimer or the null-exciton coupled species, respectively, was noticed in solution.

To shed light on the nature of the supramolecular polymers formed by Z-PDI **1**—both under kinetic and thermodynamic control—and their optical properties, we carried out a computational study that combines electronic structure calculations and a Frenkel-CT Holstein (FCTH) Hamiltonian similar to that used by Spano and co-workers^35^ and by some of us in the context of supramolecular polymers.^36^ Taking into account all of the experimental evidence obtained by the different techniques and, especially those demonstrating the formation of intermolecular H-bonding interactions and the π-stacking of the aromatic cores, we have modeled at the GFN2-xTB level different aggregated species (dimers, trimers, tetramers, and decamers) to elucidate the aggregation mode of Z-PDI **1** for both the kinetic *AggI*_*H*_ and the thermodynamic *AggII*_*n*_ aggregate. Initially, two different dimers (*Dec1* and *Dec2*) were constructed and fully optimized (Figures S9 and S10a). In both cases, the dimers are stabilized via two intermolecular H-bonds between the amide functional groups (distances of around 1.9 Å) reinforced by the π-stacking of the Z-PDI cores at ∼3.5 Å, the main difference between them being the spatial orientation of the aromatic cores. Whereas in *D1* the two Z-PDI monomers are rotated in a helical-like arrangement with a pitch angle of ∼23°, in *D2* they interact via a ladder-like pattern. In terms of energetics, the slipped π-stacked *D2* dimer is significantly more stable than the rotated *D1* dimer by 12.3 kcal mol^–1^. Based on the supramolecular organization of *Dec1* and *Dec2*, we built up and fully optimized oligomers of increasing size of Z-PDI **1** as potential models of aggregates *AggI*_*H*_ and *AggII*_*n*_ (Figure S10). The supramolecular helical- (Figure 5b) or ladder-like (Figure 5e) arrangement is preserved in the oligomers, the relative stability of the latter notably rising with the size of the oligomer (Figure S10). Specifically, the ladder-like decamer *Dec2* is found to be 124.7 kcal mol^–1^ more stable than the helical-like decamer *Dec1*. These findings suggest that the thermodynamically controlled supramolecular polymer *AggII*_*n*_ would exhibit a slipped ladder-like self-assembly, whereas a helical-like organization would be associated with the kinetically controlled supramolecular polymer *AggI_H_*.

Based on the previous structural models, the absorption spectra of the molecularly dissolved species of Z-PDI **1** and the supramolecular aggregates *AggI*_*H*_ and *AggII*_*n*_ were modeled (Figure 5a,d) using a FCTH Hamiltonian, which was parametrized based on density functional theory (DFT) and time-dependent DFT (TDDFT) calculations of monomeric/dimeric models, and a multistate diabatization method (see the Supporting Information for full details).^37^ The simulated absorption spectrum for the monomeric species clearly exhibits a marked vibrational structure for the lowest-energy S_0_ → S_1_ electronic excitation with the vibronic transitions A_0–0_, A_0–1_, A_0–2_, and A_0–3_ decreasing in intensity (Figure 5a,d).^38^ This absorption pattern coincides with that experimentally registered for **1** in CHCl_3_, and in MCH or MCH/Tol 1/1 solutions at 90 °C, ascribable to the monomeric species (Figures 3a and 4a). The absorption spectrum simulated for the ladder-type arrangement (Figure 5d) presents a pattern similar to that obtained for the monomer but with a bathochromic shift of the lowest-energy vibronic peaks (508, 470, and 448 nm) (Figure 5d).

**Figure 5:**
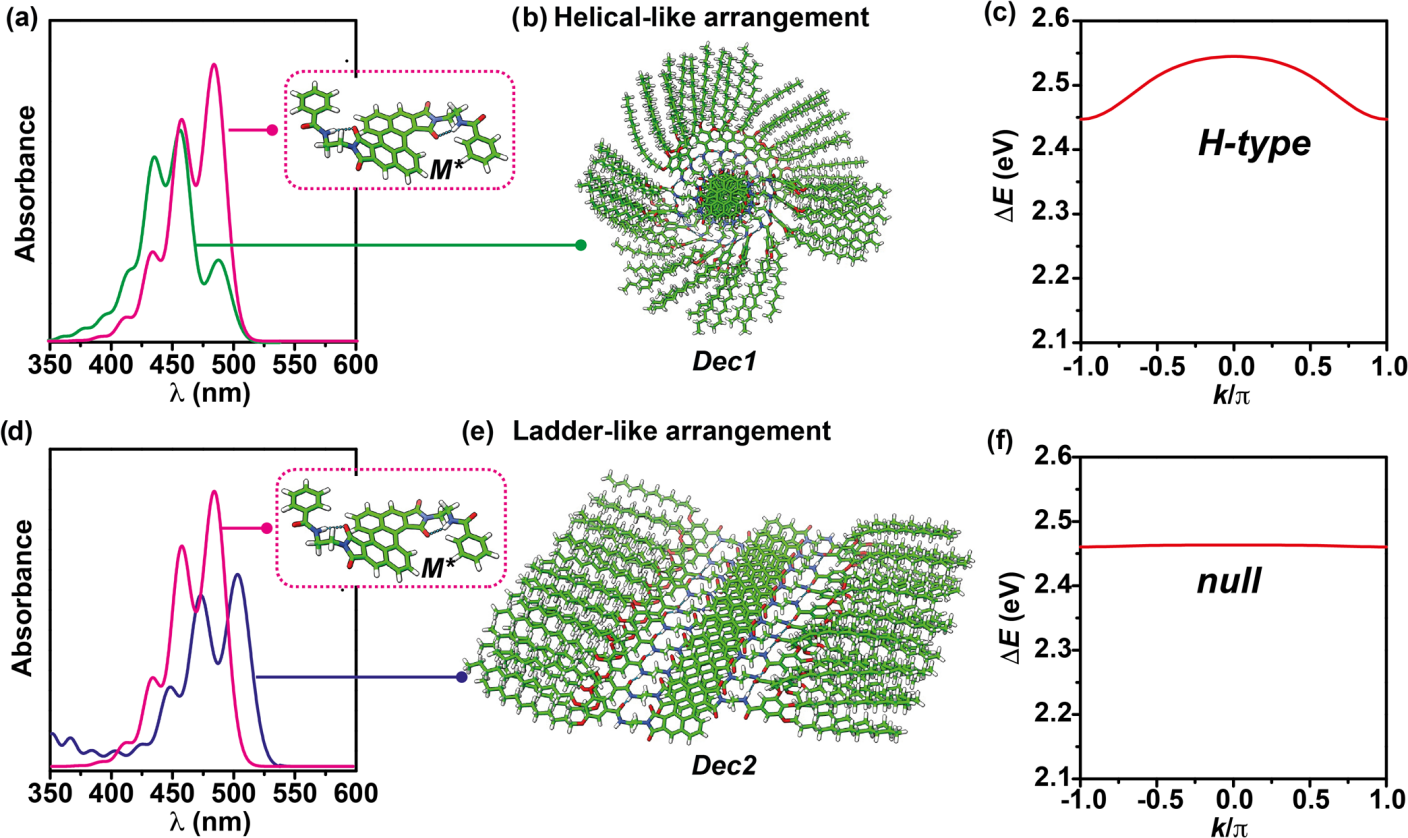
Simulated UV–vis spectra (a, d) and GFN2-xTB-optimized structures of the metastable monomer M* and the helical-like *Dec1* (b) and ladder-like *Dec2* (d) decamers (b, e). The pink spectra in parts (a, d) show the UV–vis spectrum calculated for M*. The green (a) and blue (d) spectra show the UV–vis spectra calculated for aggregates *AggI*_*H*_ and *AggII*_*n*_, respectively. Parts (c) and (f) show the dispersion for the lowest-energy vibronic band.

This is in good agreement with that experimentally observed for the thermodynamically controlled *AggII*_*n*_ aggregate (Figures 3a and 4a, blue spectra) and supports the theoretical assignment of the most stable slipped arrangement to the *AggII*_*n*_ species. In contrast, the absorption spectra computed for the helical-like arrangement (Figure 5b) show a remarkable change in terms of the intensity of the vibronic bands, with a significant increase of intensity for the vibronic A_0–2_ and A_0–1_ bands compared to the A_0–0_ peak (Figure 5a). These spectral features are in good accord with those experimentally observed for the kinetically controlled *AggI*_*H*_ aggregate (Figure 4a, green spectrum) and are generally associated with a *H*-type supramolecular aggregate.^35^

To further understand the absorption spectra predicted for *AggI*_*H*_ and *AggII*_*n*_ (Figure 5) and the nature of these aggregates, we analyzed in detail the energy position of the CT states, the different excitonic/electronic couplings, and the dispersion of the lowest-energy band (see the Supporting Information for further details). For both *AggI*_*H*_ and *AggII*_*n*_ (Figure S18 and Table S2), the lowest CT singlet excited states are predicted to be above in energy (∼0.3 to 0.4 eV) relative to the localized (Frenkel) excited states. In terms of couplings, the Coulombic excitonic interactions are relevant for *AggI*_*H*_ with a significant *J*_Coul_ coupling value of 0.070 eV, which is higher and similar in magnitude to the short-range *J*_CT_ couplings (governed by the hole/electron transfer integrals *t*_h_/*t*_e_, see Table S2). The sign and magnitude of the estimated couplings point out that *AggI*_*H*_ can be seen as a *H*-type aggregate in line with a positive dispersion of the lowest-energy vibronic band (Figures 5c and S11a). In contrast, CT-mediated interactions become more relevant for the slipped aggregate *AggII*_*n*_ in which the *J*_CT_ couplings (*t*_h_/*t*_e_) appear in the –(0.120–0.140) eV range. These couplings are of opposite sign and higher than those estimated for Coulombic interactions (*J*_Coul_ = 0.057 eV). Based on these *t*_h_/*t*_e_ and *J*_Coul_ couplings, and according to the Spano’s notation,^18^ *AggII*_*n*_ can be classified as a *HJ*-type aggregate or even a *null* aggregate if a destructive interference occurs with the consequent dispersionless band. Figures 5f (inset) and S11b show almost no dispersion for the lowest-energy vibronic band of *AggII*_*n*_, characteristic of a null aggregate.^18^

We are aware that TDDFT has difficulties in predicting the energy position of CT states even when long-range corrected density functionals are used. Nevertheless, the energy positions of the CT states can strongly shape the absorption spectra. In this regard, we have slightly varied the energy position of the CT states and the *J*_CT_ couplings (*t*_h_ and *t*_e_) predicted from the TDDFT calculations in a plausible range for *AggII*_*n*_ to see the effect on the absorption spectra (Figures S12–S14). In all of the explored situations, the simulated absorption spectra that best match the experimental spectrum registered for *AggII*_*n*_ are those that correspond to almost null aggregates with small dispersion bands (Figures S12c, S13b,c, and S14b). Therefore, all of these theoretical findings suggest that *AggII*_*n*_ is a null aggregate.

### Kinetic Studies and Living Supramolecular Polymerization

The self-assembly of Z-PDI **1** can be biased by achieving complete disassembly of the aggregated species and applying different cooling rates to reach either the kinetic or the thermodynamic aggregated states (Figure 4a). To further investigate the pathway complexity and the kinetics of the *AggI*_*H*_ → *AggII*_*n*_ transformation, we have performed a kinetic study by using a 1/1 MCH/Tol solution at *c*_*T*_ = 100 μM. Under these conditions, and applying different cooling rates, it is possible to achieve both the kinetically and thermodynamically controlled aggregated species. As stated before, a cooling rate of 0.1 °C min^–1^ yields the thermodynamically controlled *AggII*_*n*_ species that does not experience any kinetic evolution upon 24 h at 20 °C (Figure 6a). On the contrary, the application of a cooling rate of 1 °C min^–1^ yields the kinetically controlled supramolecular polymer *AggI*_*H*_ that does not evolve at room temperature upon 24 h (Figure 6a), thus demonstrating the slow *AggI*_*H*_ → *AggII*_*n*_ conversion kinetics. To accelerate this transformation, we have applied mechanical stirring, a strategy successfully utilized to speed up the kinetic conversion of supramolecular polymerizations exhibiting pathway complexity.^6c^ Mechanically stirring the solution at 400 rpm accelerates and completes the conversion of the kinetically aggregated species *AggI*_*H*_ into the thermodynamically controlled *AggII*_*n*_ in only 6 h (Figure S15).

**Figure 6:**
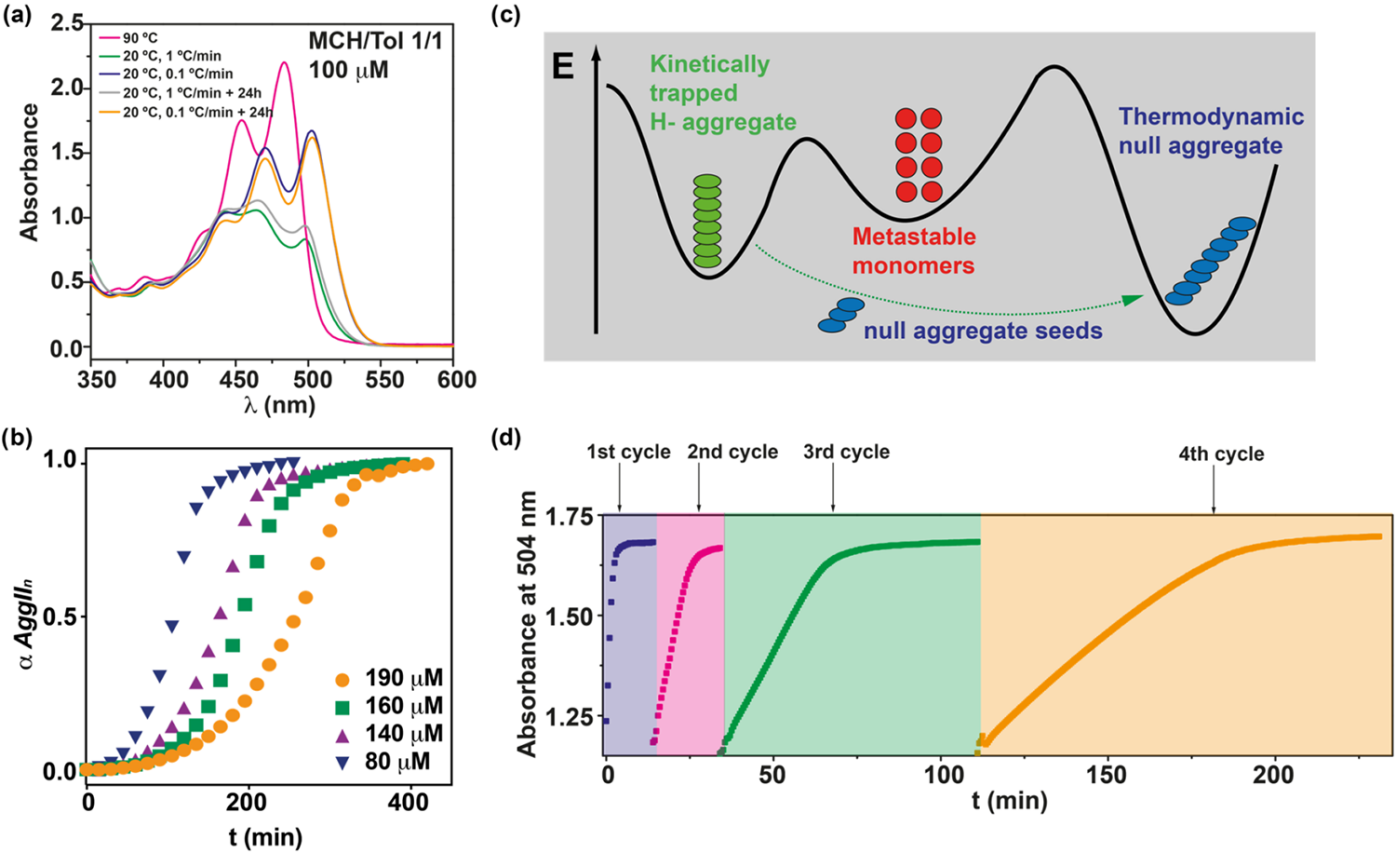
(a) UV–vis spectra of the monomeric and the two aggregated species *AggI*_*H*_ and *AggII*_*n*_ at different cooling rates and time intervals. (b) Time course transformation of the off-pathway *AggI*_*H*_ into the on-pathway *AggII*_*n*_ at different concentrations. (c) Energy landscape of the kinetically controlled supramolecular polymerization of Z-PDI **1** and the acceleration of the *AggI*_*H*_ → *AggII*_*n*_ conversion by the addition of *AggII*_*n*_ seeds. (d) Time course of the change in absorption at 504 nm while *AggII*_*n*_ seeds are diluted with the *AggI*_*H*_ species, showing repeated polymerization on each addition of *AggI*_*H*_ but with increasingly slower rates. In all cases, the *AggI*_*H*_ → *AggII*_*n*_ conversion is conducted in a MCH/Tol 1/1 mixture and stimulated by mechanical stirring (400 rpm).

To further explore the relationship between the two polymerization pathways, the kinetic evolution of the *AggI*_*H*_ → *AggII*_*n*_ transformation was monitored at different concentrations. For this study, solutions of Z-PDI **1** in MCH/Tol 1/1 were first heated to 90 °C to ensure complete disassembly and then rapidly cooled to 25 °C to induce formation of *H*-type *AggI*_*H*_ (Figure S16). UV–vis spectra were recorded at 25 °C under mechanical stirring at 400 rpm until the complete transformation of *H*-type aggregates into null aggregates was observed (Figures 6b and S16). These experiments reveal that the *AggI*_*H*_ → *AggII*_*n*_ conversion is accelerated at lower concentrations, indicative of a competitive mechanism (Figure 6c). In this mechanism, both *AggI*_*H*_ and *AggII*_*n*_ compete for the free monomeric species, *AggI*_*H*_ representing the off-pathway product and *AggII*_*n*_ the on-pathway ensemble (Figure 6c).^5^ Interestingly, the kinetic profiles present a sigmoidal shape (Figure 6b), diagnostic of an autocatalytic process that consists in a kinetically prevented nucleation, followed by accelerating growth processes.^30,39a,39d^

Finally, we have also demonstrated the living character of the *AggI*_*H*_ → *AggII*_*n*_ conversion by applying the methodology firstly described by Sugiyasu, Takeuchi and co-workers, in which different kinetic cycles are performed by adding the thermodynamically controlled *AggII*_*n*_ as active seeds to a solution of the kinetically controlled *AggI*_*H*_ (Figure S17).^6c^ This strategy has been utilized to carry out the living supramolecular polymerization of referable systems.^39^ Interestingly, the addition of *AggII*_*n*_ seeds to a solution of *AggI*_*H*_ notably accelerates the *AggI*_*H*_ → *AggII*_*n*_ conversion that is completed in only 10 min (Figure 6d). In this first cycle of the living supramolecular polymerization, equal volumes of the solution of both aggregated species at the same concentration were utilized (Figure S17). Using this 1/1 vol/vol solution, three additional cycles were performed by adding equal volumes of a solution of the kinetically controlled *AggI*_*H*_ species. The kinetic profiles of these additional cycles show that the kinetic evolution is slowed by half due to the dilution effect on the active termini produced by the consecutive additions of the seeds (Figure 6d). The living character of the process was also corroborated by registering AFM images of the different cycles that show a proportional length of the fibrillar aggregates depending on the cycle. Thus, the seeds appear as short fibers that grow upon successive seeding cycles until reaching very long fibrillar aggregates of tens of micrometers long (Figure S18).

## Conclusions

Herein, the synthesis of Z-shaped PDI **1** and its self-assembling features are reported. The lateral tris­(dodecyloxy)­benzamide units of **1** facilitate the formation of metastable monomeric units (M*) that enable a kinetically controlled supramolecular polymerization. This supramolecular polymerization presents a competitive pathway complexity, in which two different aggregates (*AggI*_*H*_ and *AggII*_*n*_) are formed. The synergy between the experimental evidence and the theoretical calculations demonstrates that the kinetically controlled *AggI*_*H*_ aggregated species corresponds to a helical arrangement governed by the π-stacking of the aromatic backbones, reinforced by intermolecular H-bonding interactions between the amide functional groups. This aggregate can be seen as an *H*-type aggregate with dominant long-range excitonic interactions of Coulombic character. Under adequate experimental conditions (MCH solution or slow cooling down of a MCH/Tol 1/1 solution), Z-PDI **1** leads to the thermodynamically controlled *AggII*_*n*_ aggregated species, which is theoretically predicted to be significantly more stable and presents a ladder-type structure. The self-assembling and optical features of *AggII*_*n*_ suggest the formation of a *null* aggregate in which both the Coulombic and the CT interactions are relevant but of different sign. The conversion of the kinetically aggregated species into the thermodynamically aggregated supramolecular polymer is made possible by simple mechanical stirring or by carrying out living supramolecular polymerization. The studies presented herein demonstrated that subtle adjustments of the Coulombic and charge-transfer interactions can result in the conversion of a conventional *H*-type aggregate into an unusual *null* aggregate.

## Supplementary Material



## Notes

Full experimental details, characterization and additional spectroscopic measurements, additional AFM images, additional theoretical details and supporting references including Figures S1–S17 (PDF)

## Abbreviations

PDIs: perylenediimides
*J*_Coul_: Coulombic coupling
*J*_CT_: charge-transfer-mediated coupling
CT: charge transfer
KC: kinetically controlled
TC: thermodynamically controlled
*M**: metastable monomeric units
MCH: methylcyclohexane
Tol: toluene
HOPG: highly oriented pyrolytic graphite
SD: solvent denaturation
EQ: equilibrium
ACQ: aggregation caused quenching
DFT: density functional theory
TDDFT: time-dependent density functional theory
