# Supplementary material for: Unraveling Pathway Complexity in the Supramolecular Polymerization of Z‑Shaped Perylenediimides: From Kinetic H‑Aggregates to Thermodynamic Null Supramolecular Polymers
Source: J Am Chem Soc. 2025 Jul 7;147(28):25024–34. doi: 10.1021/jacs.5c08436 (PMC12272694; doi:10.1021/jacs.5c08436)
Supplement: Supplementary file 1 [file ja5c08436_si_001.pdf]

Supporting Information

Unraveling Pathway Complexity in the Supramolecular Polymerization of Z-Shaped Perylenediimides: From Kinetic H-Aggregates to Thermodynamic Null Supramolecular Polymers

Alfonso J. Schwalb,[a] Cristina Naranjo,[a] Alberto Fernández-Alarcón,[b] Fátima García,[a] Enrique Ortí,[b] Juan Aragó,[b]* and Luis Sánchez[a]*

[a] *Departamento de Química Orgánica, Facultad de Ciencias Químicas, Universidad Complutense de Madrid, E-28040 Madrid, Spain.*

[b]*Instituto de Ciencia Molecular (ICMol), Universitat de València, 46980 Paterna, Spain*

Contents:

1. Experimental Section S-1

2. Synthetic Details and Characterization S-2

3. Collection of Spectra S-4

4. Supplementary Figures and Tables S-6

Synthetic scheme S-6

VT-¹H NMR experiments S-6

Optimized geometries of the monomeric species S-7

Concentration dependent ¹H NMR experiments S-7

UV-Vis spectra of ***1*** *in MCH* S-9

AFM images of AggII S-8

VT-UV-Vis experiments S-9

AFM images of AggI S-10

Optimized geometries of the n-mers S-11

Simulated UV-Vis spectra of ***1*** S-12

UV-Vis at different times by applying mechanical stirring S-14

Schematic illustration of the living supramolecular polymerization S-15

AFM images of the living supramolecular polymerization S-15

5. Quantum Yield Determination S-16

6. Theoretical Calculations S-17

7. References S-22

1. Experimental Section

General. All solvents were dried according to standard procedures. Reagents were used as purchased. All air-sensitive reactions were carried out under argon atmosphere. Flash chromatography was performed using silica gel (Merck, Kieselgel 60, 230-240 mesh or Scharlau 60, 230-240 mesh). Analytical thin-layer chromatography (TLC) was performed using aluminium-coated Merck Kieselgel 60 F254 plates. NMR spectra were recorded on a Bruker Avance 300 MHz (^{1}H: 300 MHz; ^{13}C: 75 MHz) spectrometer at 25 °C using partially deuterated solvents as internal standards. Coupling constants (*J*) are denoted in Hz and chemical shifts (δ) in ppm. Multiplicities are denoted as follows: s = singlet, d = doublet, t = triplet, q = quartet, quin = quintuplet, m = multiplet, and br = broad. FTIR spectra were recorded on a Bruker Tensor 27 (ATR device) spectrometer. FTIR spectra in film were recorded on a Jasco FT-IR4600 spectrometer using a CaF_2 cell with a path length of 0.1 nm. UV-Vis spectra were registered on a Jasco-V630 spectrophotometer equipped with a Peltier thermoelectric temperature controller. The freshly prepared solutions were measured and, after that, the samples were heated up to 90 °C. The samples at 90 °C were registered and cooled to 20 °C. Emission spectra were recorded on a Perkin-Elmer LS55 spectrophotometer. Atomic force microscopy (AFM) images were taken on a SPM Nanoscope IIIa multimode microscope working on tapping mode with a TESPA-V2 tip (Veeco) at a working frequency of ~235 kHz. High-resolution mass spectra (HRMS) were recorded on a MALDI Bruker daltonics Ultraflex TOF/TOF spectrometer.

2. Synthetic Details and Characterization

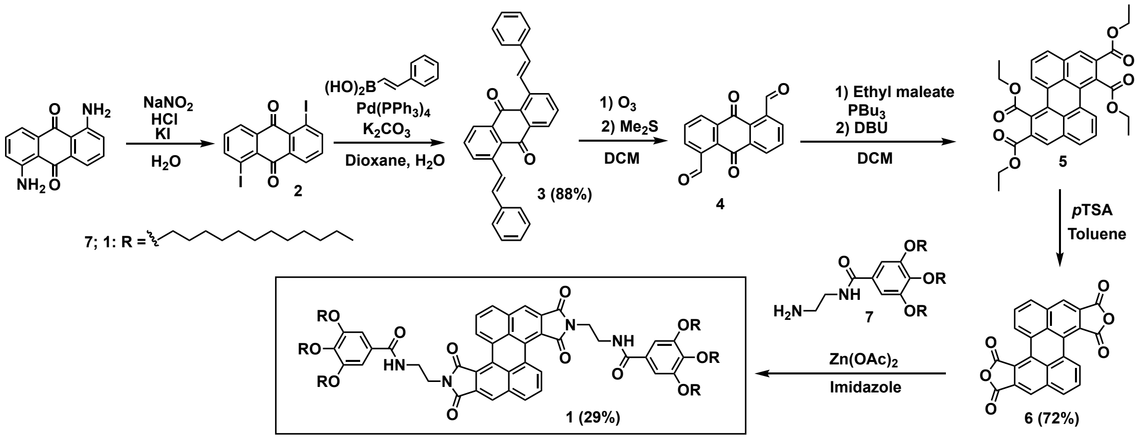

Scheme S1. Synthesis of Z-PDI **1**.

Compounds **2**,[S1] **4**,[S2] **5**,[S2] and **7**[S3] were prepared according to reported synthetic procedures and showed identical spectroscopic properties. Compound **3** was prepared through a modified synthetic procedure showing identical spectroscopic properties.[S2]

1**,5-di((*****E*****)-styryl)anthracene-9,10-dione (3).**[S2] 1,5-diiodo-9,10-anthraquinone (**2**) (0.100 g, 0.2 mmol, 1.0 equiv.) (*E*)-styrylboronic acid (0.067 g, 0.4 mmol, 2.1 equiv.), tetrakis(triphenylphosphine)palladium(0) (0.012 g, 0.01 mmol, 5 mol%), and potassium carbonate (0.138 g, 1.3 mmol, 6.0 equiv.) were added to a round bottom flask under argon atmosphere. A mixture of 7.5 mL of dioxane and 2 mL of water were purged with argon for 1 hour and added to the reaction flask and the reaction mixture was heated at 92 °C for 18 hours. Then, the reaction was cooled to room temperature, washed with water (80 mL), extracted with CH_2Cl_2 (80 mL), dried with $MgSO_4$ and the solvent removed under vacuum. The crude was purified by column chromatography (silica, CH_2Cl_2/hexane 1/1) to obtain a yellow solid (0.079 g). Yield: 88%. ^{1}H NMR (300 MHz,$CDCl_3$) δ: 8.37 (d, 2H, J = 16.1 Hz), 8.28 (dd, 2H, J = 7.7 Hz, 1.3), 7.98 (dd, 2H, J = 7.9, 1.4 Hz), 7.75 (td, 2H, J = 7.8, 0.6 Hz), 7.65 (d, 4H, J = 7.5 Hz), 7.42 (m, 4H), 7.33 (d, 2H, J = 7.2 Hz), 7.07 (d, 2H, J = 16.2 Hz).

Peryleno[1,2-c:7,8-c']difuran-1,3,8,10-tetraone (6). Tetraester **5** (0.100 g, 0.09 mmol, 1 equiv.) and *p*-toluenesulfonic acid (0.172 g, 0.4 mmol, 5.0 equiv.) were dissolved in toluene (3 mL) and heated at 100 °C for 30 h. The reaction mixture was then cooled to room temperature and centrifuged with methanol (3 x 6 mL) to obtain the final product as an orange powder (0.052 g). The extremely low solubility of the target dianhydride in the most common solvents prevented registering NMR spectra and compound **6** was utilized without any further purification. Yield: 72%. HRMS-MALDI-TOF m/z calcd. for $C_{24}H_8O_6$ $[M]^+$ 392.0321, found 392.0305.

***N,N'*-((1,3,8,10-tetraoxo-1,3,8,10-tetrahydroanthra[9,1-ef:10,5-e'f']diisoindole-2,9-diyl)bis(ethane-2,1-diyl))bis(3,4,5-tris(dodecyloxy)benzamide) (1)**

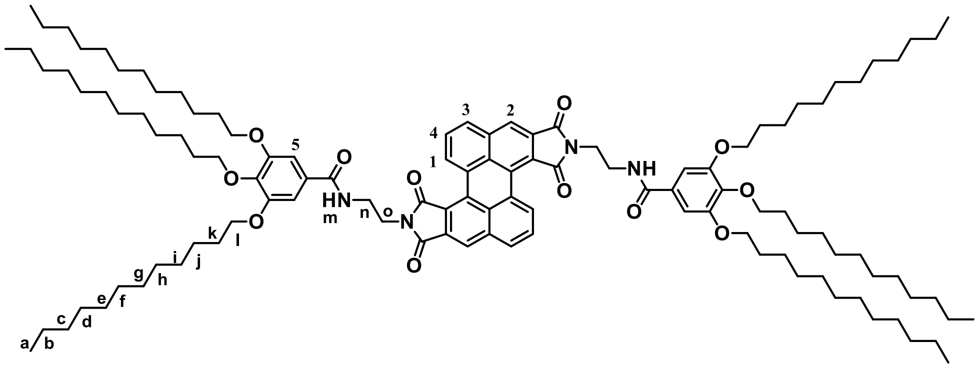

Compound **6** (0.030 g, 0.07 mmol, 1.0 equiv.), benzamide **7** (0.325 g, 0.16 mmol, 2.5 equiv.), zinc acetate (0.014 g, 0.07 mmol, 1.0 eq.), and imidazole (0.3 g) were purged with argon in a microwave sealed tube. The reaction mixture was reacted for 40 minutes at 165 °C under microwave irradiation. The obtained crude was washed with brine (30 mL) and extracted with CH_2Cl_2 (30 mL). The organic layer was dried with $MgSO_4$ and the solvent removed under vacuum. The crude was purified by column chromatography (silica, CH_2Cl_2/EtOAc/MeOH 97/2/1) to obtain an orange solid (0.022 g). The product was further purified by centrifugation in methanol (3 x 6 mL). Yield: 29%. ^{1}H NMR (300 MHz,$CDCl_3$) δ: 9.02 (dd, 2H, H_1, J = 7.7, 1.1 Hz), 8.25 (s, 2H, H_2), 8.02 (dd, 2H, H_3, J = 7.7, 1.1 Hz), 7.73 (dd, 2H, H_4, J = 7.9 Hz), 6.99 (s, 4H, H_5), 6.86 (t, 4H, H_m, J = 5.1 Hz), 4.09 (m, 4H, H_n), 3.96 (m, 12H, H_l), 3.81 (m, 4H, H_o), 1.75 (m, 12H, H_k), 1.42 (m, 12H, H_j), 1.35–1.19 (m, 96H, $H_{b\text{-}i}$), 0.87 (m, 18H, H_a). ^{13}C NMR (75 MHz, $CDCl_3$) δ: 168.6, 167.8, 167.7, 153.2, 141.1, 134.7, 133.8, 133.1, 132.6, 132.4, 130.2, 129.2, 129.1, 128.5, 122.9, 105.7, 77.2, 73.6, 69.3, 40.1, 38.2, 32.1, 32.1, 30.5, 29.9, 29.8, 29.8, 29.7, 29.6, 29.5, 26.3, 26.2, 22.8, 14.3, 1.2. HRMS-MALDI-TOF m/z calcd. for $C_{114}H_{172}N_4O_{12}$ $[M]^+$ 1789.2972, found 1789.3010.

3. Collection of Spectra

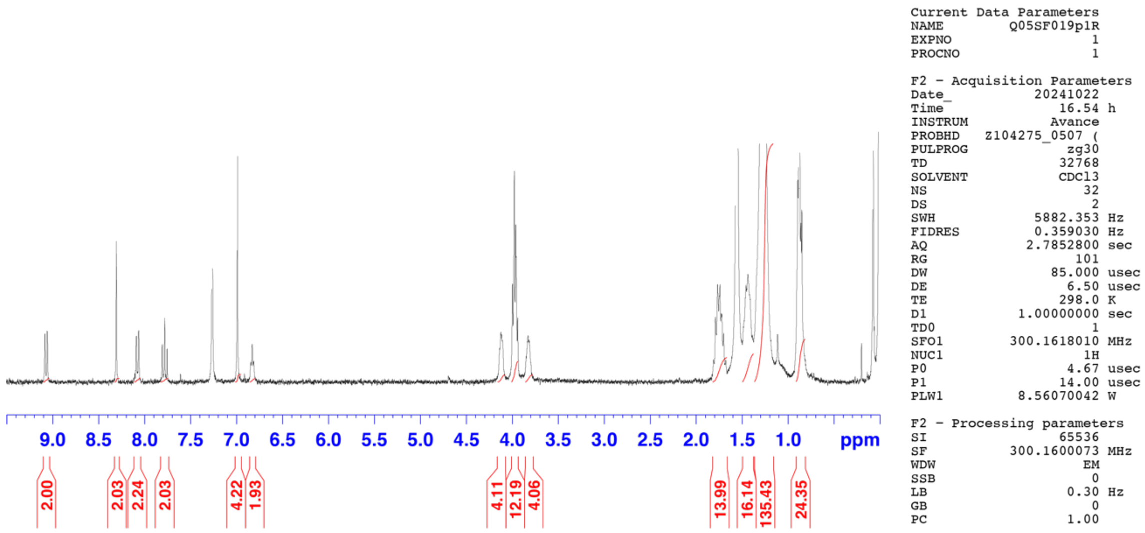

^{1}H NMR ($CDCl_3$, 300 MHz, 298 K) of compound **1**.

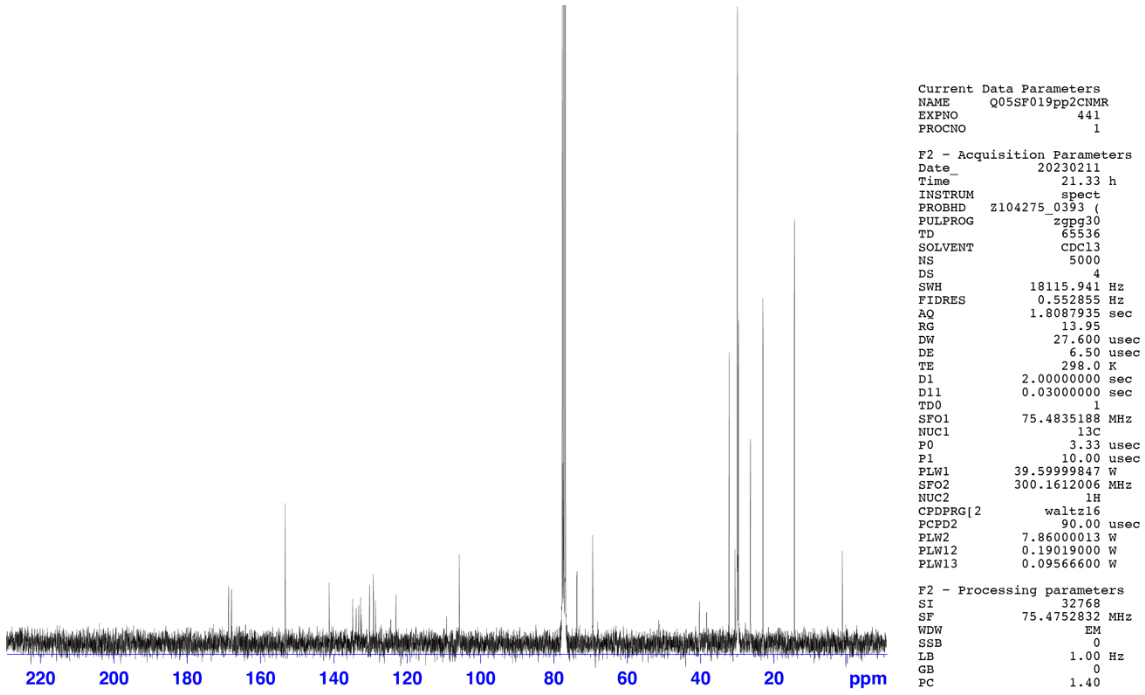

^{13}C NMR ($CDCl_3$, 75 MHz, 298 K) of compound **1**.

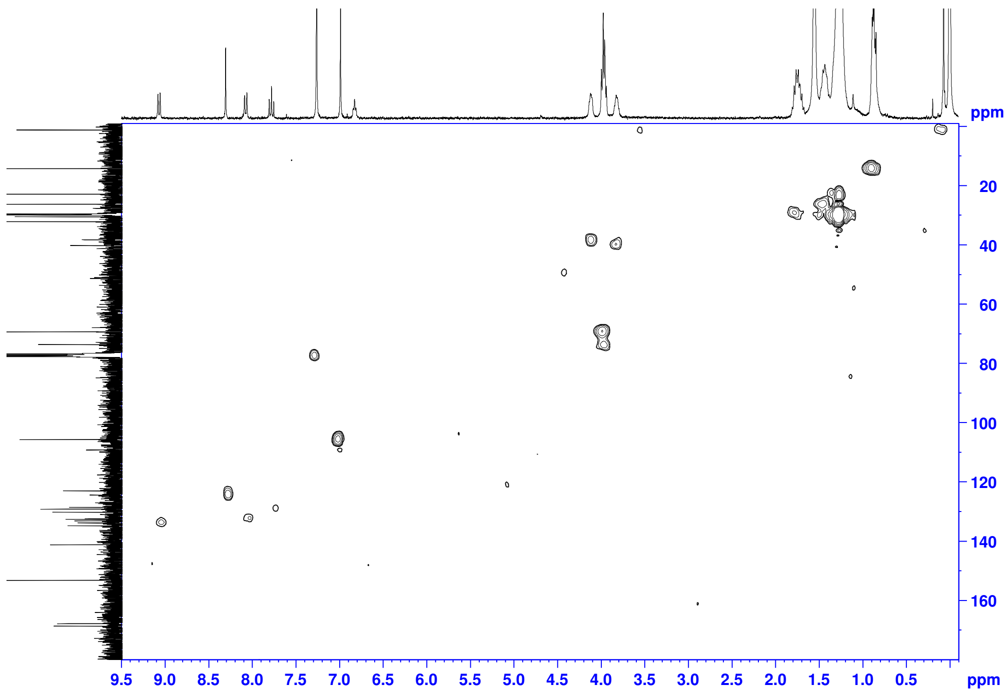

^{1}H,^{13}C-HMQC spectrum ($CDCl_3$, 298 K) of compound **1**.

4. Supplementary Figures and Tables

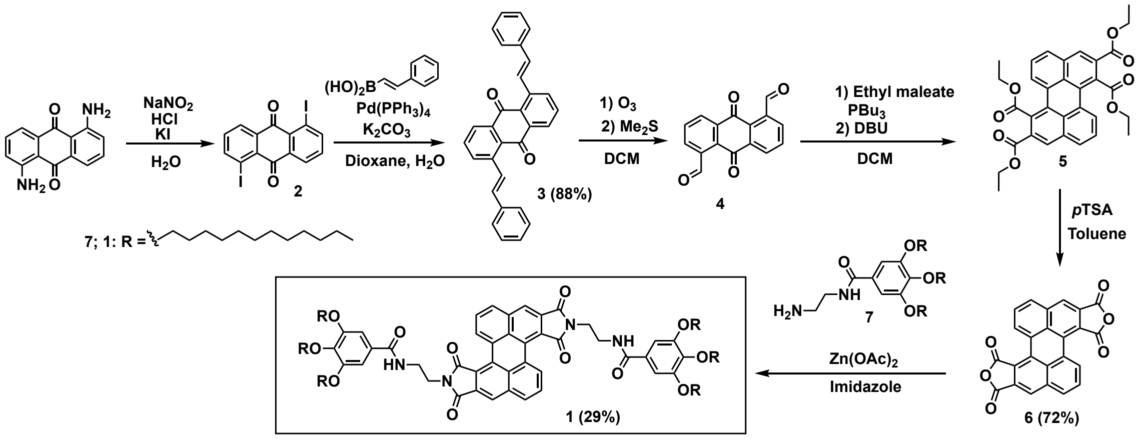

Scheme S1. Synthesis of the Z-PDI **1**.

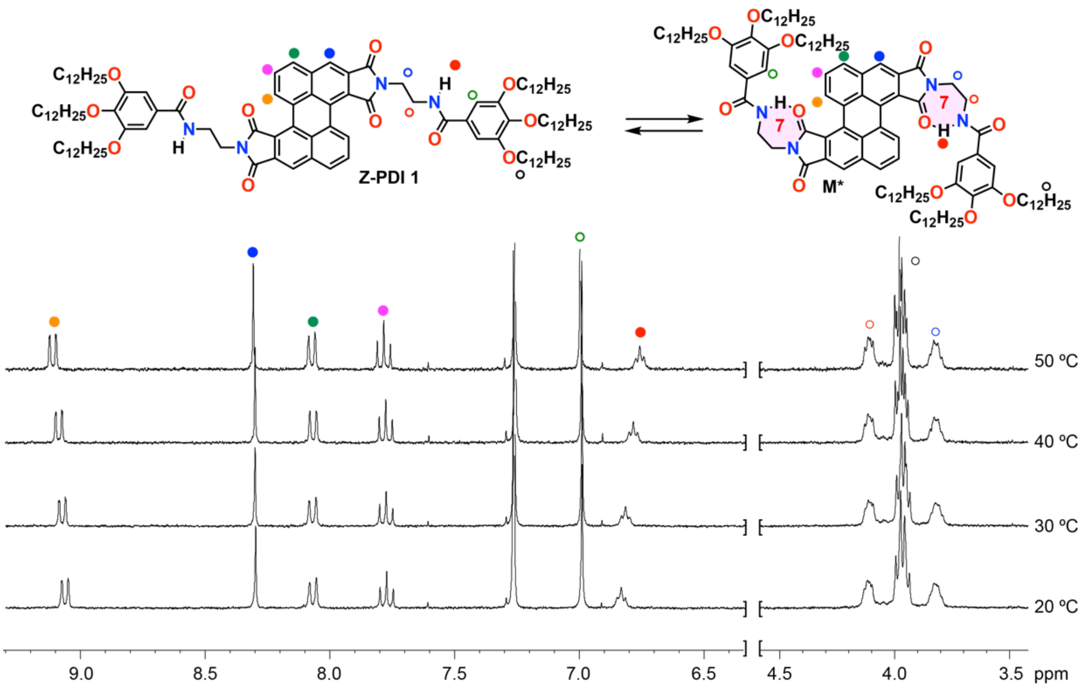

Figure S1. Partial ^{1}H NMR spectra of Z-PDI **1** recorded at different temperatures showing the aromatic and some of the aliphatic protons ($CDCl_3$; 300 MHz; c_T =1 mM, 298 K).

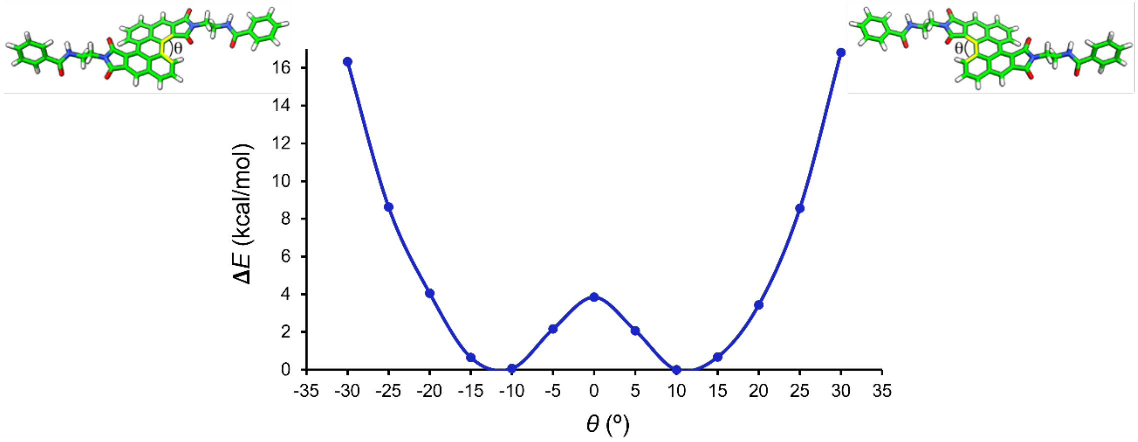

Figure S2. Optimized geometry of the atropisomers formed upon the torsion of the central perylene core of Z-PDI **1** and energetic profile of the racemization of these configurations calculated at the GFN2-xTB level. Yellow bonds in the modelled geometries depict the torsion angle θ.

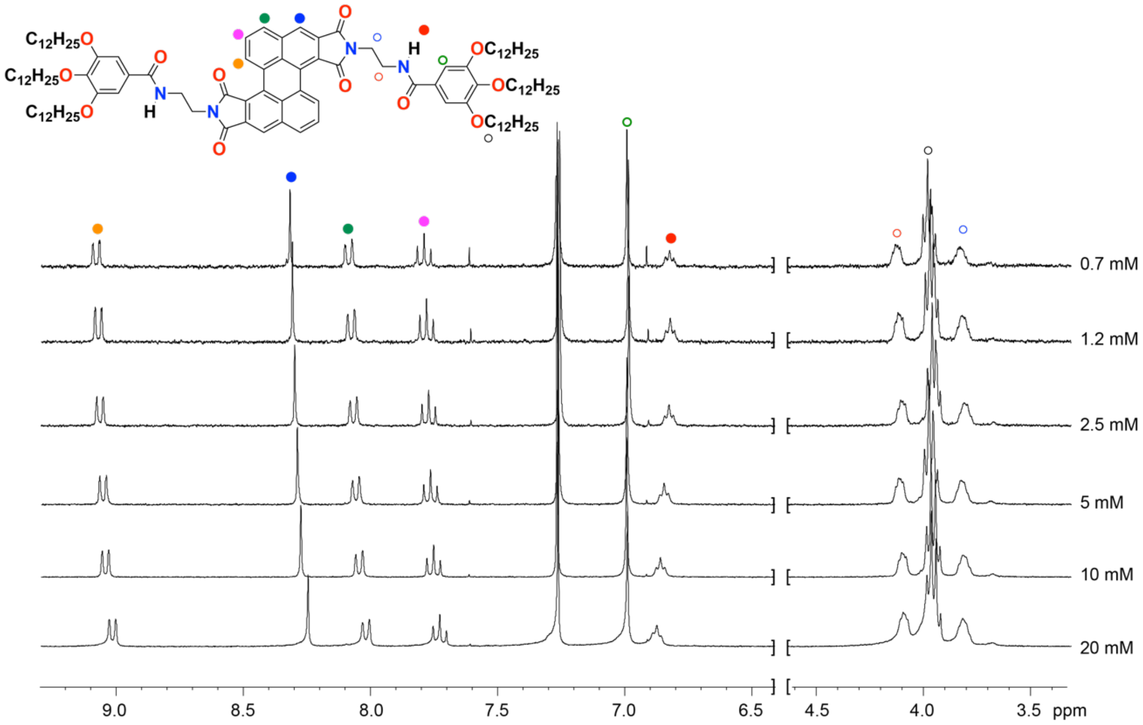

Figure S3. Partial ^{1}H NMR spectra of Z-PDI **1** recorded at different concentrations showing the aromatic and some of the aliphatic protons ($CDCl_3$; 300 MHz; 298 K).

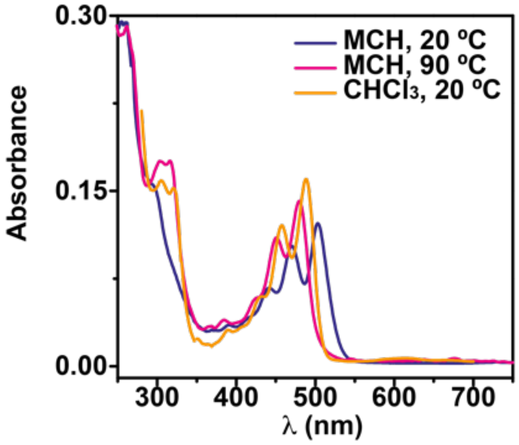

Figure S4. UV-Vis spectra of Z-PDI **1** in $CHCl_3$ and MCH at c_T = 10 µM.

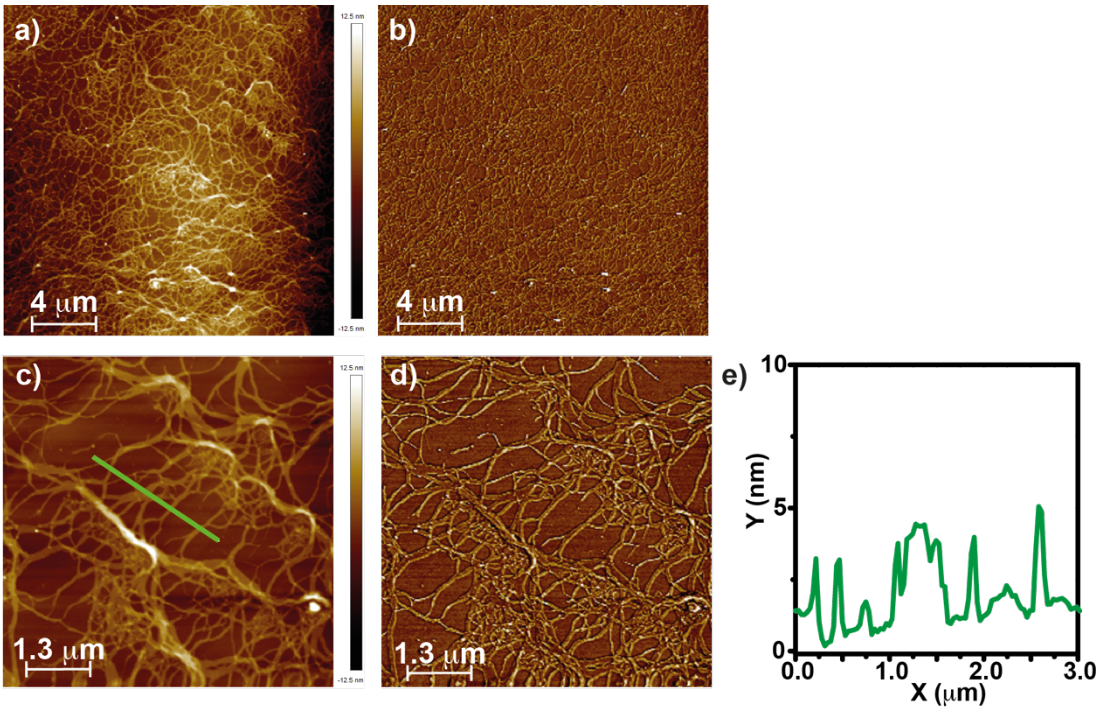

Figure S5. Height (a, c) and phase (b, d) AFM images of the fibrillar aggregates formed from Z-PDI **1** in MCH (HOPG; c_T = 10 µM; 20 °C). Panel (e) shows the height profile of the fibers along the green line in panel (c).

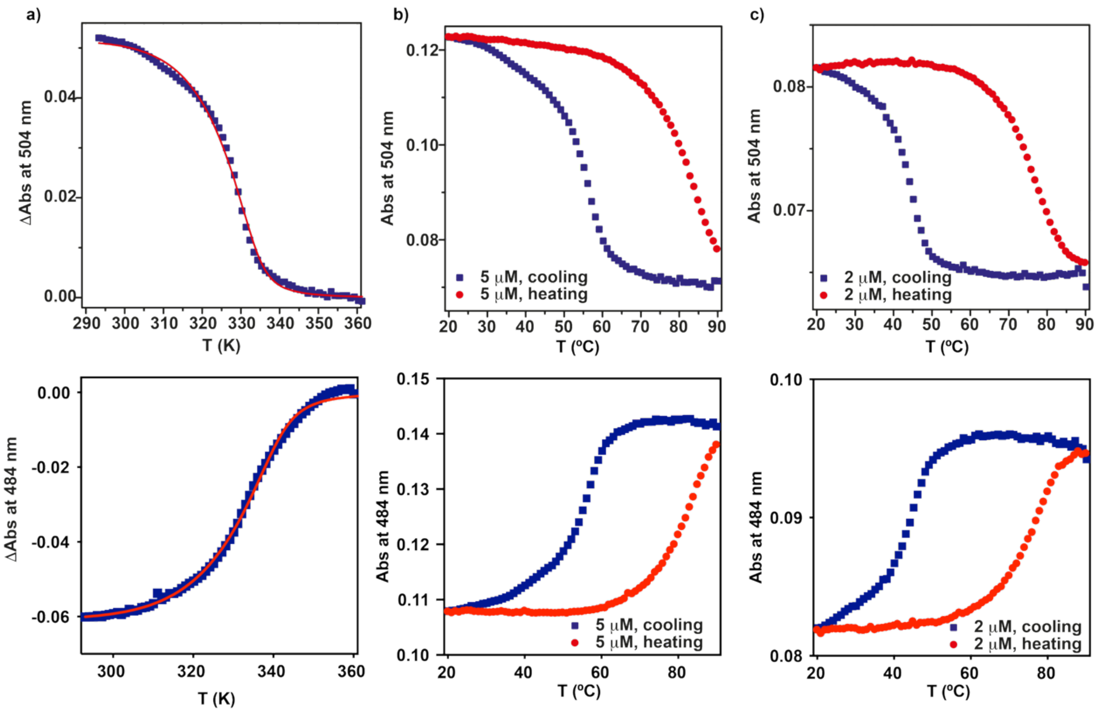

Figure S6. (a) Cooling curves obtained by plotting the changes in the absorbance at 504 nm (upper part) and 484 nm (bottom part) versus the temperature in MCH as solvent and at c_T = 10 µM. The red line depicts the fitting of this variation to the one-component EQ model. (b, c) Cooling and heating curves in MCH at 5 *µ*M (b) and 2 *µ*M (c) using a cooling or heating rate of 1 ºC/min and monitoring the absorbance at 504 nm (upper part) and 484 nm (bottom part).

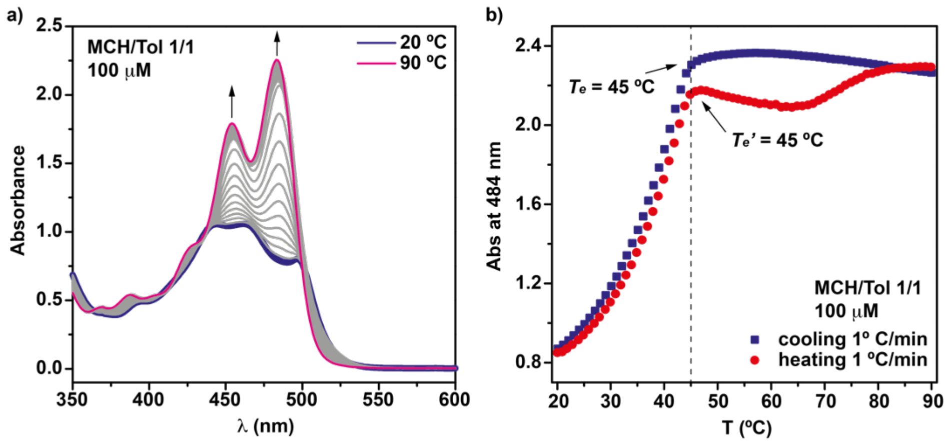

Figure S7. (a) UV-Vis spectra of **1** dissolved in a MCH/Tol 1/1 mixture at c_T = 100 µM and recorded at different temperatures applying a heating rate of 1 ºC/min. The arrows indicate the changes in the UV-Vis spectra upon heating the solution. (b) Heating and cooling curves obtained by plotting the changes in the absorbance at 484 nm versus temperature.

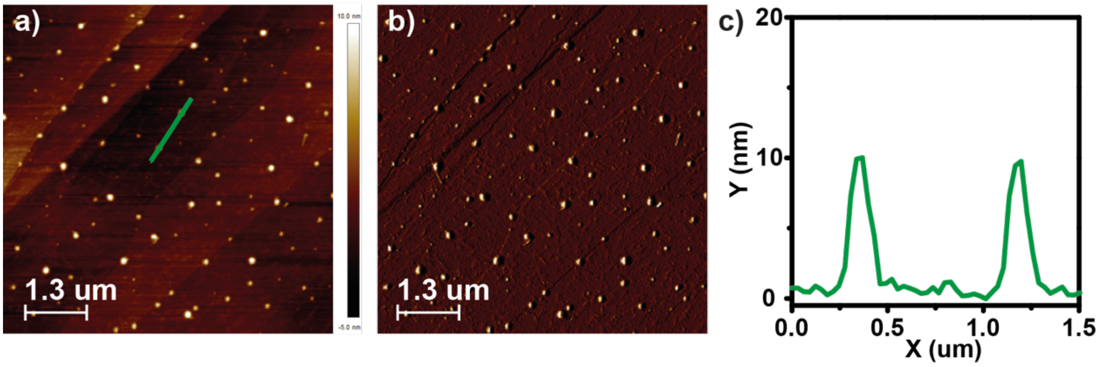

Figure S8. Height (a) and phase (b) AFM images of the nanoparticles formed from Z-PDI **1** in MCH/Tol 1/1 (HOPG; c_T = 10 µM; 20 °C). Panel (c) shows the height profile of the nanoparticles along the green line in panel (a).

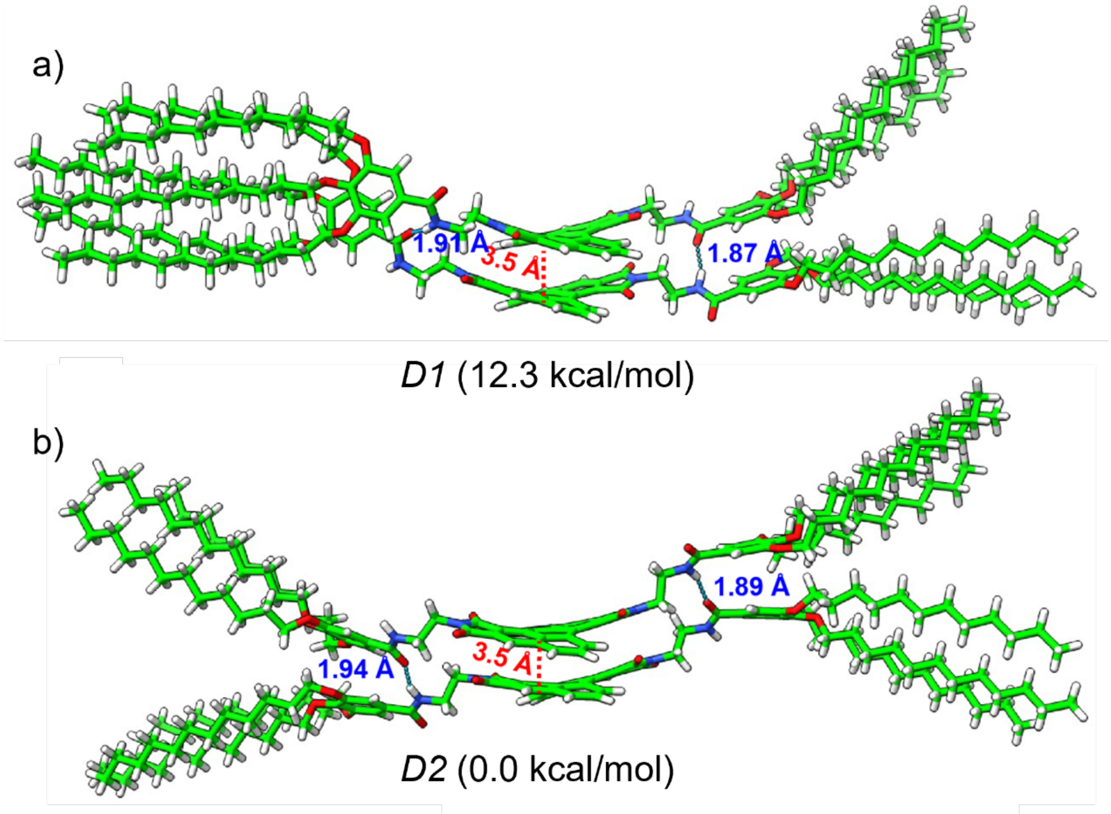

Figure S9. GFN2-xTB-optimized geometries calculated for the helical-like *D1* (a) and the slipped ladder-like *D2* (b) dimers of Z-PDI **1** shown in a side view. The relative energies and the distances of the intermolecular H-bonding interactions (in blue) and the π-stacking of the aromatic backbones (in red) are denoted.

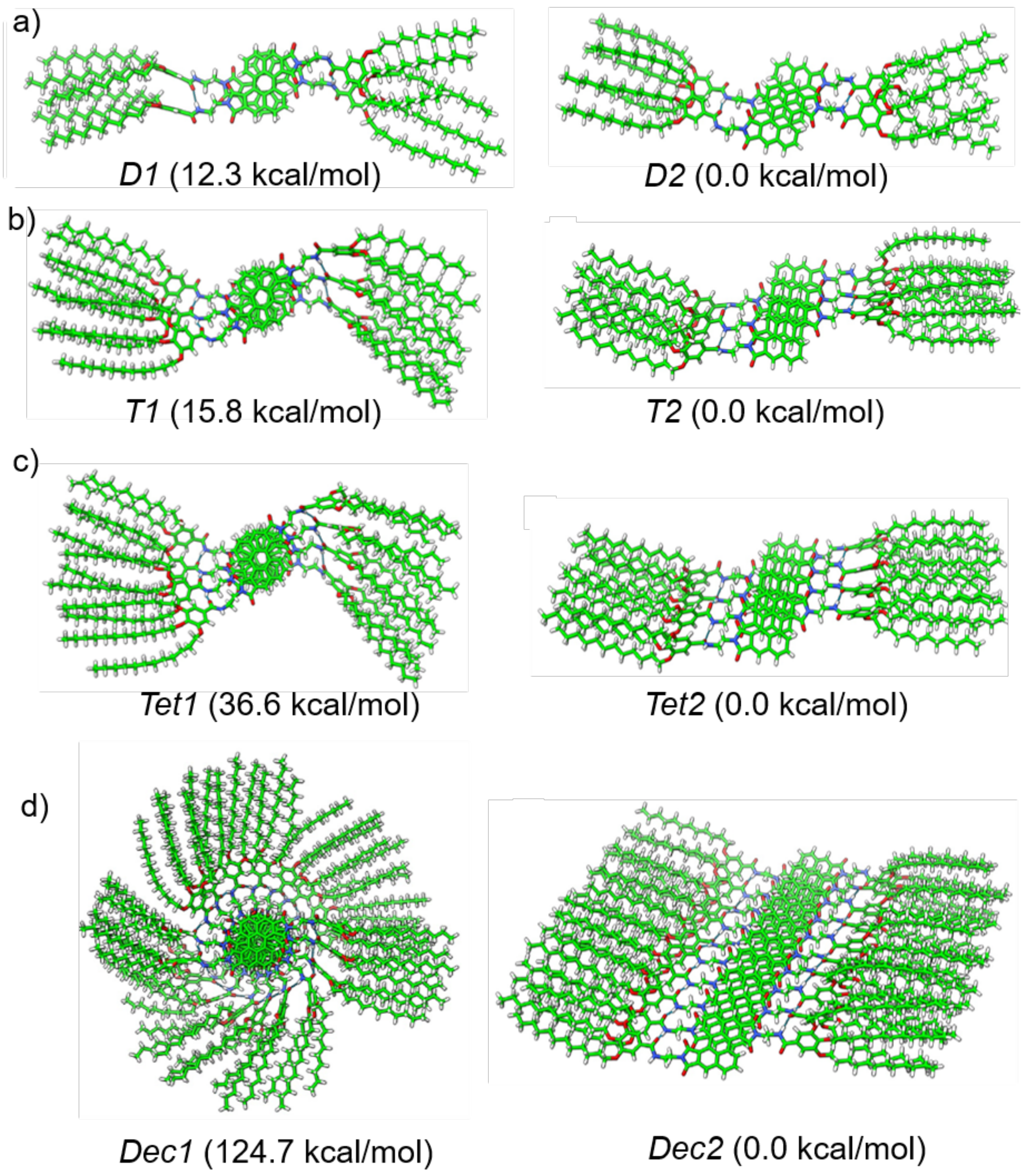

Figure S10. GFN2-xTB-optimized geometries calculated for helical-like (left) and slipped ladder-like (right) Z-PDI **1** oligomers of increasing size: dimers (a), trimers (b), tetramers (c), and decamers (d) shown in a top view. Relative energies are given within parentheses.

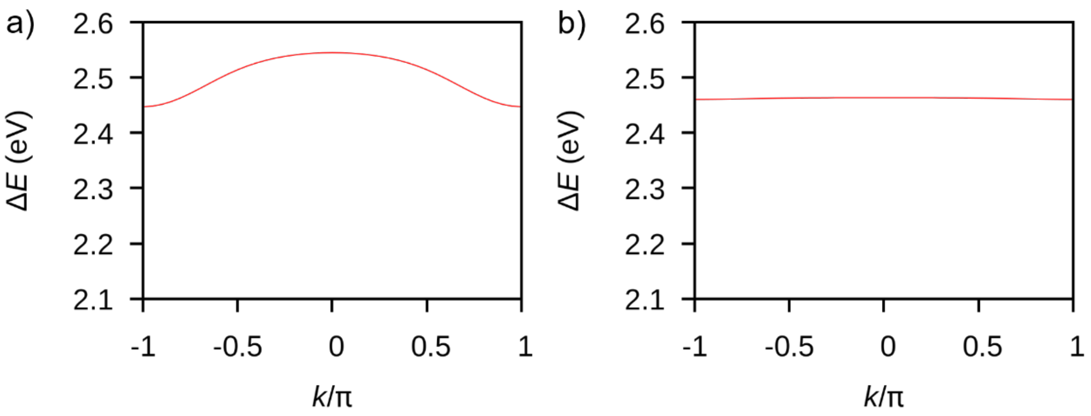

Figure S11. Diagrams of the lowest-energy vibronic band calculated with the FCTH Hamiltonian for *AggI*$_H$ (a) and *AggII*$_n$ (b) with the same set of parameters used for the simulated absorption spectrum of Figure 5 (Table S3).

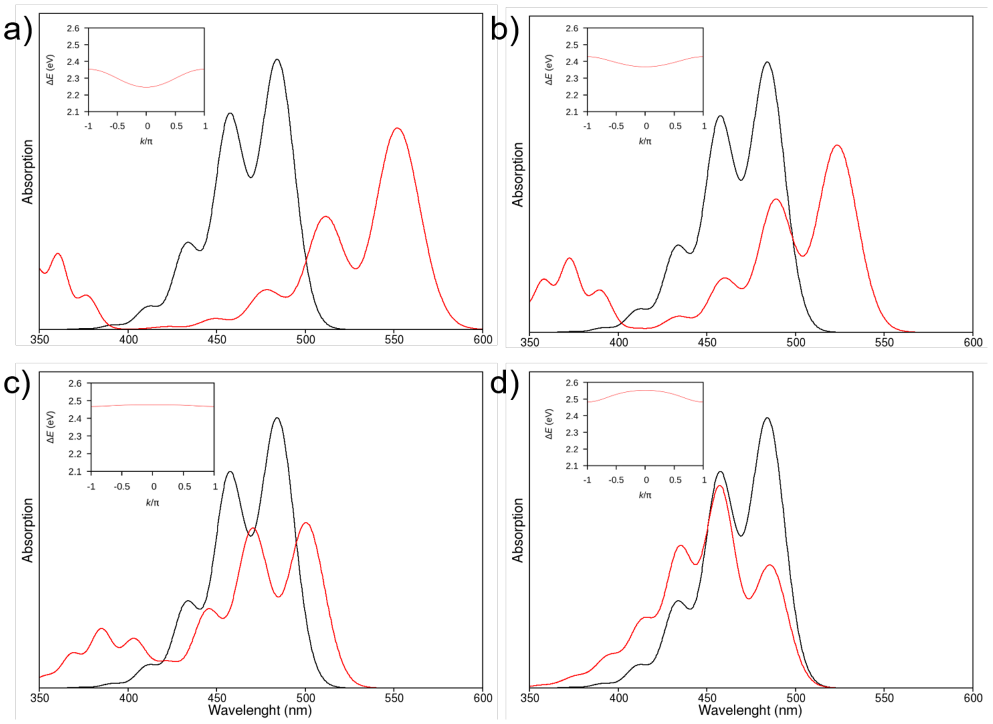

Figure S12. Simulated UV-Vis spectra calculated for the monomer (black line) and *AggII*$_n$ (red line) using the parameters of Table S4, Δ_{CT} = 0.3 eV, and $t_h = t_e$ values of –0.200 (a), –0.150 (b), –0.100 (c), and –0.050 eV (d). Insets display the profile calculated for the lowest-energy vibronic band for *AggII*$_n$.

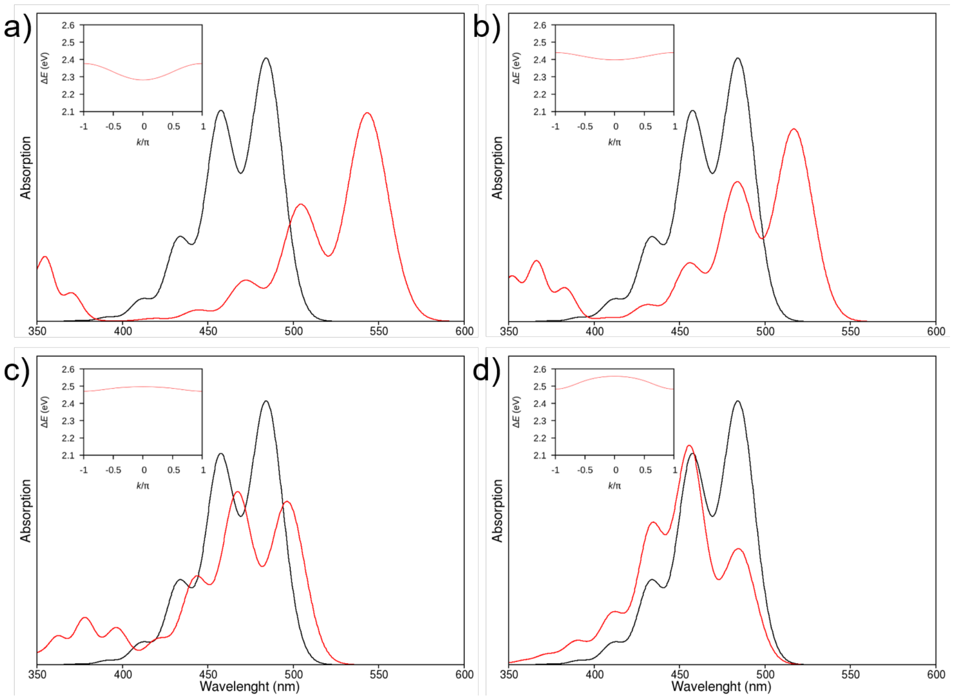

Figure S13. Simulated UV-Vis spectra calculated for the monomer (black line) and *AggII$_n$* (red line) using the parameters of Table S4, Δ_{CT} = 0.4 eV, and $t_h = t_e$ values of –0.200 (a), –0.150 (b), –0.100 (c), and –0.050 eV (d). Insets display the profile calculated for the diagram of the lowest-energy vibronic band for *AggII$_n$*.

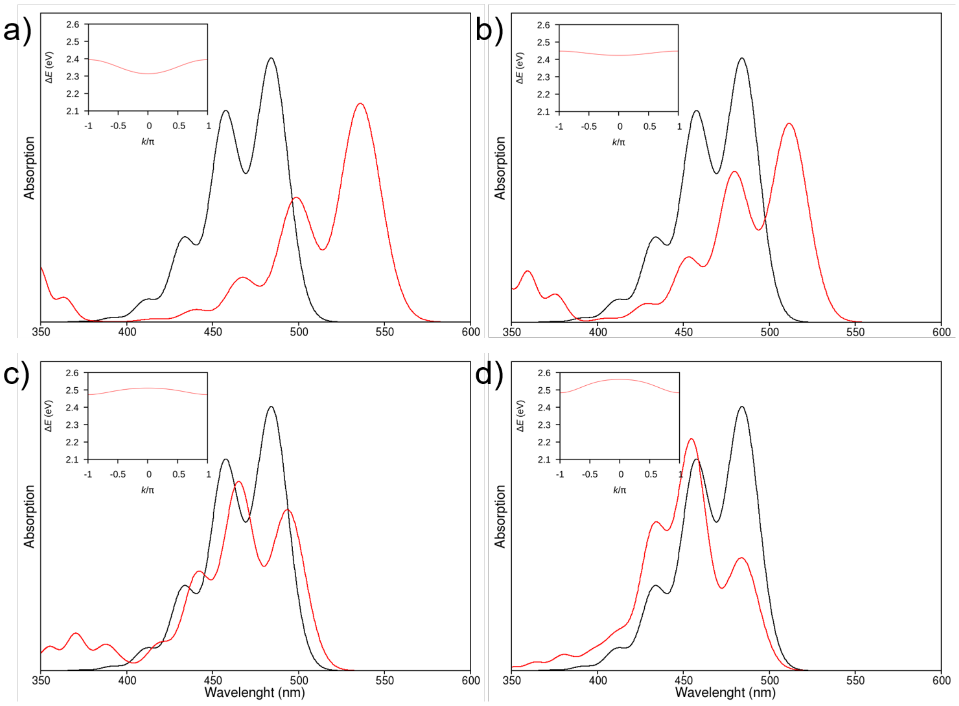

Figure S14. Simulated UV-Vis spectra calculated for the monomer (black line) and *AggII$_n$* (red line) using the parameters of Table S4, Δ_{CT} = 0.5 eV, and $t_h = t_e$ values of –0.200 (a), –0.150 (b), –0.100 (c), and –0.050 eV (d). Insets display the profile calculated for the lowest-energy vibronic band for *AggII$_n$*.

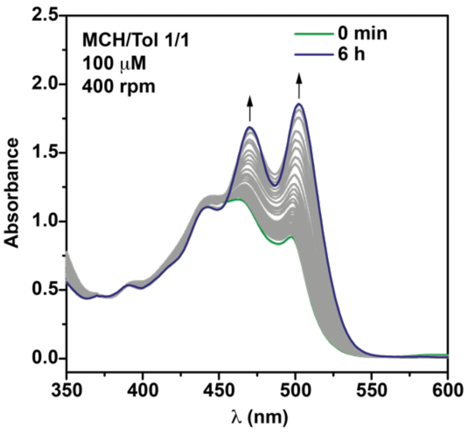

Figure S15. UV-Vis spectra of Z-PDI **1** at different time intervals registered by applying mechanical stirring (400 rpm; 1/1 MCH/Tol; c_T = 100 μM).

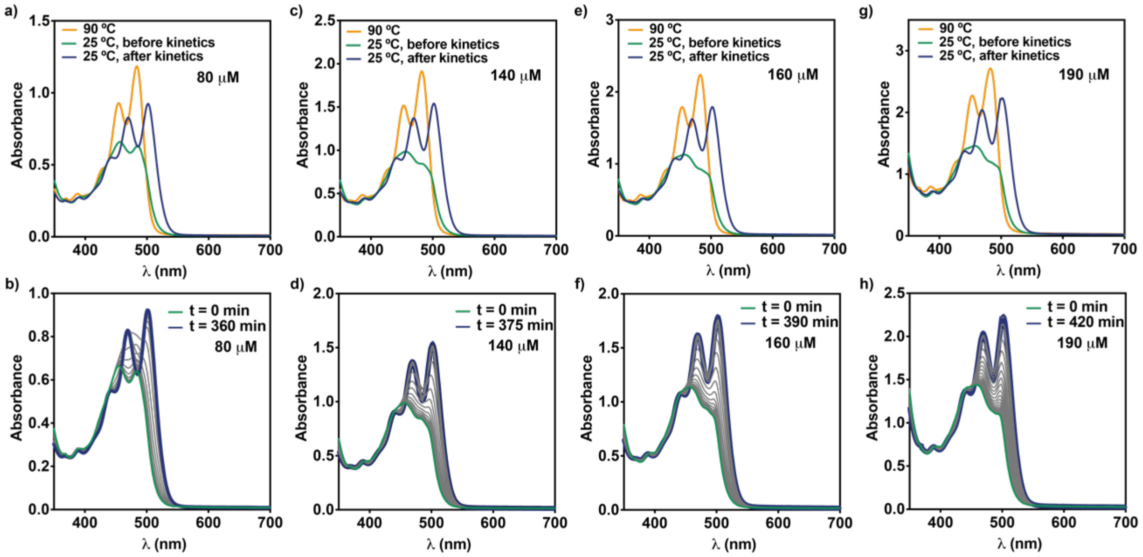

Figure S16. UV-Vis spectra of Z-PDI **1** registered at different concentrations, temperatures, and time intervals by applying a mechanical stirring (400 rpm; 1/1 MCH/Tol; c_T = 100 μM). Panels (a), (c), (e), and (g) show the UV-Vis spectra recorded at 90 ºC (ascribable to the monomeric species) and at 25 ºC before (green line) and after (blue line) the kinetic study. Panels (b), (d), (f) and (h) show the UV-Vis spectra at 25 ºC with intervals of 15 min.

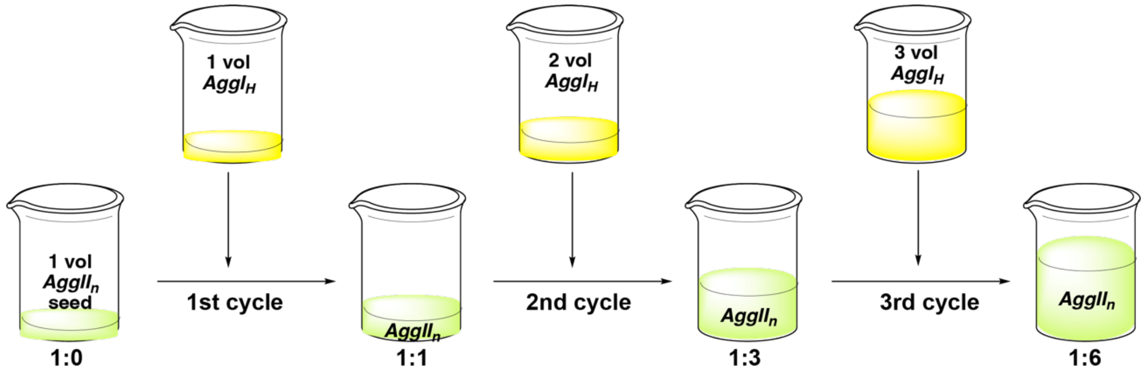

Figure S17. Schematic illustration of the living supramolecular polymerization experienced by Z-PDI **1**. The sonication of a solution of $AggII_n$ for 2 minutes generates the active seeds. Successive aliquots with increasing volume of the H-type $AggI_H$ are added to the $AggII_n$ solution and the kinetic profile is monitored at 504 nm, an absorption wavelength characteristic of the thermodynamically controlled $AggII_n$. Experimental conditions: MCH/Tol 1/1 as solvent; c_T = 80 µM for both aggregated species; T = 25 °C.

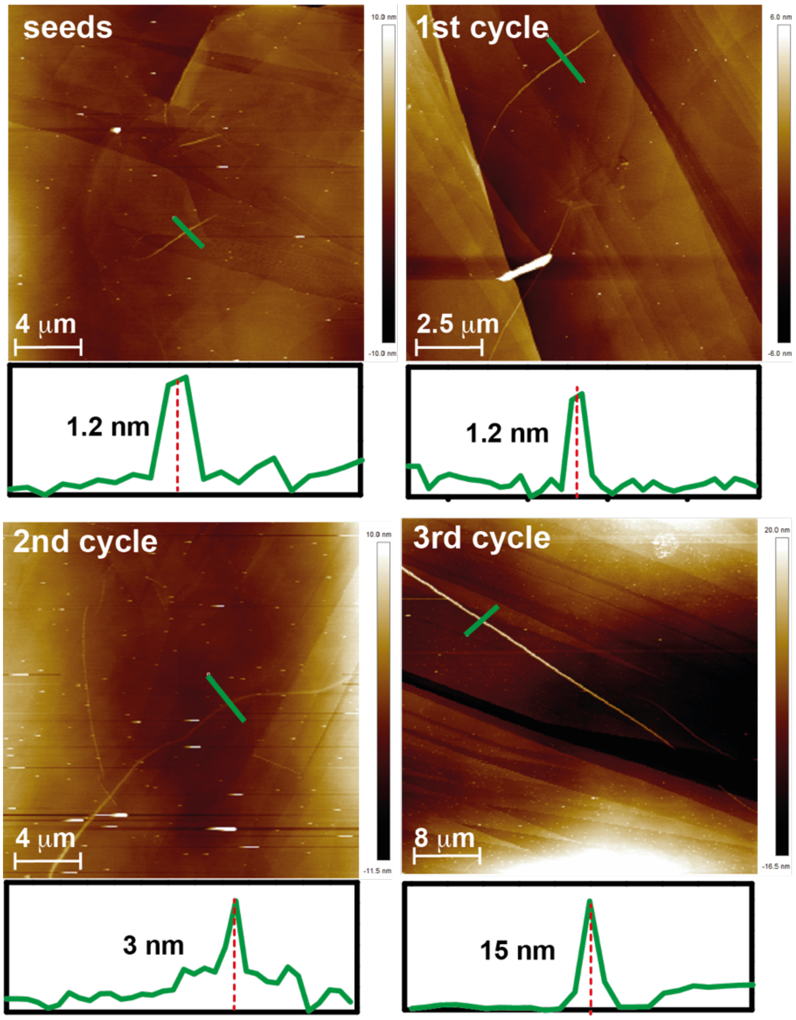

Figure S18. AFM images of the aggregates formed during the different cycles performed in the living supramolecular polymerization.

5. Quantum Yield Determination

To determine the fluorescence quantum yield, we have registered UV-Vis spectra on a Jasco-V630 spectrophotometer and the corresponding emission spectra were acquired in Jasco FP-8350 spectrofluorometer. Both instruments were equipped with a Peltier thermoelectric temperature controller. The fluorescence quantum yields were calculated by comparison with the corresponding emission spectrum of Fluorescein in basic ethanol (Φ_{fluo}= 0.97) following the formula:

$$\Phi_{fluo} = \Phi_{fluo\,ref} \frac{I}{I_{ref}} \frac{1-10^{-A_{ref}}}{1-10^{-A}} \frac{n^2}{n_{ref}^2}$$

where Φ_{fluo} is the fluorescence quantum yield of the sample, I is the integrated area of the emission intensity under the spectral curve, A is the absorbance at the excitation wavelength, and n is the refractive index of the solvent (1.446 for $CHCl_3$, 1.423 for MCH, 1.456 for MCH/Toluene 1:1, and 1.361 for basic ethanol).

Table S1. Derived quantum yields (Φ_{fluo}) for the different monomeric and aggregated species of Z-PDI **1.**

Species	Excitation λ (nm)	Solvent	Temperature (°C)	Φ_{fluo}
Kinetic aggregate	456	MCH/Toluene (1:1)	25	0.52
Monomer	453	MCH/Toluene (1:1)	90	0.67
Null aggregate	470	MCH/Toluene (1:1)	25	0.66

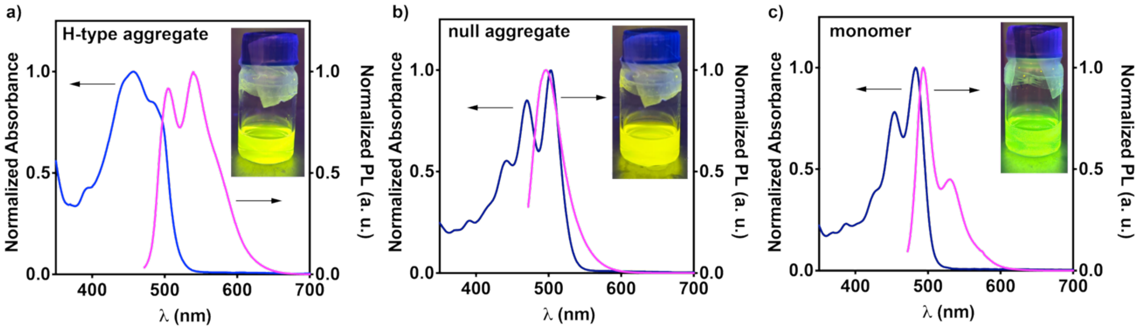

Figure S19. Normalized UV-Vis and emission spectra of the H-type aggregate (a), null aggregate (b) and monomeric species (c) formed in the mixture MCH/Tol, 1/1, at c_T = 100 μM and at 20 °C (a, b) and 90 °C (c). The insets in panels (a-c) depict a picture of the corresponding solution of **1** under irradiation at λ = 365 nm

6. Theoretical Calculations

Frenkel–charge-transfer Holstein (FCTH) Hamiltonian. The FCTH Hamiltonian used to simulate the absorption spectra of the Z-PDI **1** in the monomeric and polymeric forms can be written as:

$$\hat{H} = \hat{H}_{FE} + \hat{H}_{CT} + \hat{H}_{FE-CT} + \hat{H}_{Vib}, \qquad \text{Eq. S1}$$

where the Frenkel part in the nearest neighbor's approximation reads as:

$$\hat{H}_{FE} = \sum_{n=1}^{N} \left[\hbar\omega_{0-0} + \hbar\omega_{vib} \left(\lambda_0 \left(b_n^{\dagger} + b_n \right) + \lambda_0^2 \right) \right] |n\rangle\langle n| + \sum_{n=1}^{N-1} J_{Coul} |n+1\rangle\langle n| + h.c., \qquad \text{Eq. S2}$$

where $E_{S_1} = \hbar\omega_{0-0}$ represents the energy of a local electronic singlet excitation (Frenkel state) on the *n*th site in the aggregate, J_{Coul} is the excitonic coupling, $\hbar\omega_{vib}$ the frequency of the effective normal mode that modulates the energy of the excited sites and λ_0^2 is the Huang-Rhys (HR) factor of the Frenkel state (S_1) that measures the relative shift with respect to the ground electronic state S_0. $b_n^{\dagger}$ and b_n are the creation and annihilation operators of a vibrational quantum in the unshifted S_0 nuclear potential well on-site *n*.

Similarly, we define a CT Hamiltonian (Eq. S3), which is diagonal since we assume only interaction between consecutive sites, as:

$$\hat{H}_{CT} = \sum_{n=1}^{N-1} \left[\begin{array}{l} \left(\Delta_{CT} + \hbar\omega_{vib} \left(\lambda_+ \left(b_n^{\dagger} + b_n \right) + \lambda_- \left(b_{n+1}^{\dagger} + b_{n+1} \right) + \lambda_+^2 + \lambda_-^2 \right) |n^+, n+1^-\rangle\langle n^+, n+1^-| \right) + \\ \left(\Delta_{CT} + \hbar\omega_{vib} \left(\lambda_+ \left(b_{n+1}^{\dagger} + b_{n+1} \right) + \lambda_- \left(b_n^{\dagger} + b_n \right) + \lambda_+^2 + \lambda_-^2 \right) |n^-, n+1^+\rangle\langle n^-, n+1^+| \right) \end{array} \right], \qquad \text{Eq. S3}$$

where $\Delta_{CT} = E_{CT} - E_{S_1}$ is the energy difference between Frenkel and CT states, and λ_+^2 and λ_-^2 are the HR factors associated to the cation and anion, respectively.

Eq. S4 contains the interaction between the FE and CT states *via* the hole and electron transfer integrals (t_h and t_e), defined as the Hamiltonian coupling elements between FE and CT states with a hole or electron of difference, respectively.

$$\hat{H}_{FE-CT} = \sum_{n=1}^{N-1} \left[t_e |n\rangle\langle n^+, n+1^-| + t_h |n\rangle\langle n^-, n+1^+| \right] + h.c. \qquad \text{Eq. S4}$$

Lastly, the last term of the Hamiltonian just contains the energy of the vibrational quanta in the ground state potential energy surface (Eq. S5):

$$\hat{H}_{Vib} = \hbar\omega_{vib}\sum_{n=1}^{N} b_n^\dagger b_n \ . \qquad \text{Eq. S5}$$

The full Hamiltonian is expressed in a delocalized basis set including one- and two- particle states for FE excitons and two-particle states for CT states. Therefore, the ath eigenstate wave vector is expanded as:

$$\left|\Psi_{a,k}\right\rangle = e^{ikn}\sum_{n,v}\begin{bmatrix} c_{n,v_n}^{a,k}\left|n,v_n\right\rangle + \sum_{m\neq n,w}\left(c_{n,v_n,m,w_m}^{a,k}\left|n,v_n,m,w_m\right\rangle\right) + \\ \sum_{w}\left(c_{n^+,v_n,n+1^-,w_{n+1}}^{a,k}\left|n^+,v_n,n+1^-,w_{n+1}\right\rangle + c_{n^-,v_n,n+1^+,w_{n+1}}^{a,k}\left|n^-,v_n,n+1^+,w_{n+1}\right\rangle\right)\end{bmatrix}. \qquad \text{Eq. S6}$$

$\left|n,v_n\right\rangle$ and $\left|n,v_n,m,v_m\right\rangle$ are the local one- and two-particle FE states, where the exciton is at site *n*, and v_n and w_m are the vibrational quanta in sites *n* and *m*, respectively, and $\left|n^+,v_n,n+1^-,w_{n+1}\right\rangle$ and $\left|n^-,v_n,n+1^+,w_{n+1}\right\rangle$ are the corresponding two-particle CT states. In this contribution, we use a basis cutoff with the maximum number of quanta $\left(v_n + w_m\right)$ limited to 5. The intensity of the absorption spectra is computed as:

$$I_{abs}\left(\omega\right) = \sum_{a,k=0}\left|\mu_{0a,k=0}\right|^2 e^{-\frac{\left(\omega-\omega_{a,k=0}\right)^2}{2\sigma^2}} \ , \qquad \text{Eq. S7}$$

where $\mu_{0a,k=0}$ is the transition dipole moment between the vibrational ground state and eigenstate a at the high symmetry *k* point*,* and the exponential part adds a homogeneous broadening with a standard deviation (*σ*) of 0.05 eV. The transition dipole moments are estimated by rotating the diabatic transition dipole moments with the eigenvector matrix from diagonalizing the model Hamiltonian from Eqs. S1-S5.

Excited state calculations and diabatization. Based on the *Dec1* and *Dec2* models optimized at the GFN2-xTB level (Figure 10d), we extracted the central dimers to avoid terminal effects, removed the peripheral alkoxy chains and re-optimized the dimers at the B3LYP-D3/cc-pVTZ level[S5] (dimers *D1a* and *D1b* in Figure S18) to obtain a more accurate description of the intermolecular H-bonds and π-π contacts. These optimized structures were further simplified by removing the terminal benzenes, the amide groups and a methylene group but preserving the relative orientation of the PDI cores obtained from the previous optimizations (dimers *D2a* and *D2b* in Figure S19). Over the *D1b* and *D2b* simplified models, we performed TDDFT calculations using the long-range corrected *ω*B97X-D[S6] density functional and the cc-pVDZ basis set.[S7] The long-range corrected *ω*B97X-D functional was selected for its superiority for the description of CT states.[S8] All these calculations have been performed with the Gaussian 16 program package.[S9]

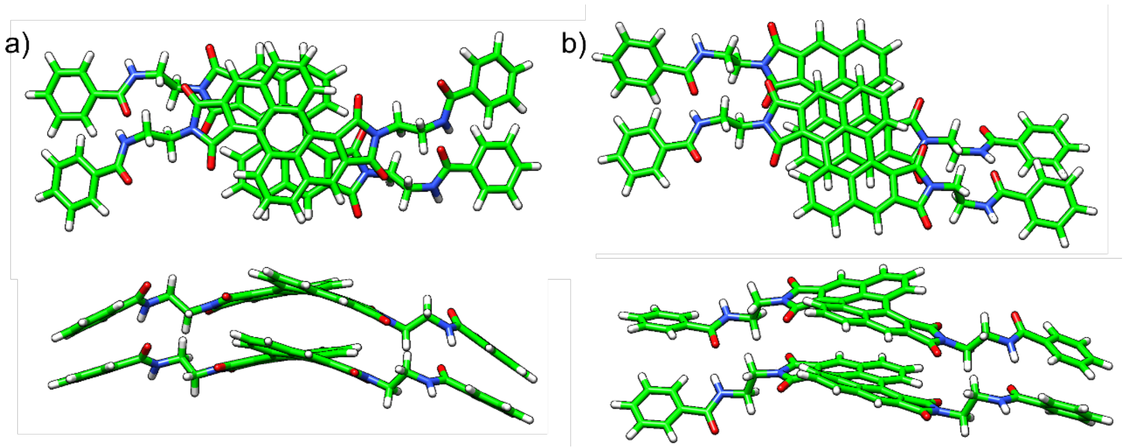

Figure S18. Top and side views of the B3LYP-D3/cc-pVTZ-optimized structures computed for the dimeric *D1a* (a) and *D2a* (b) models.

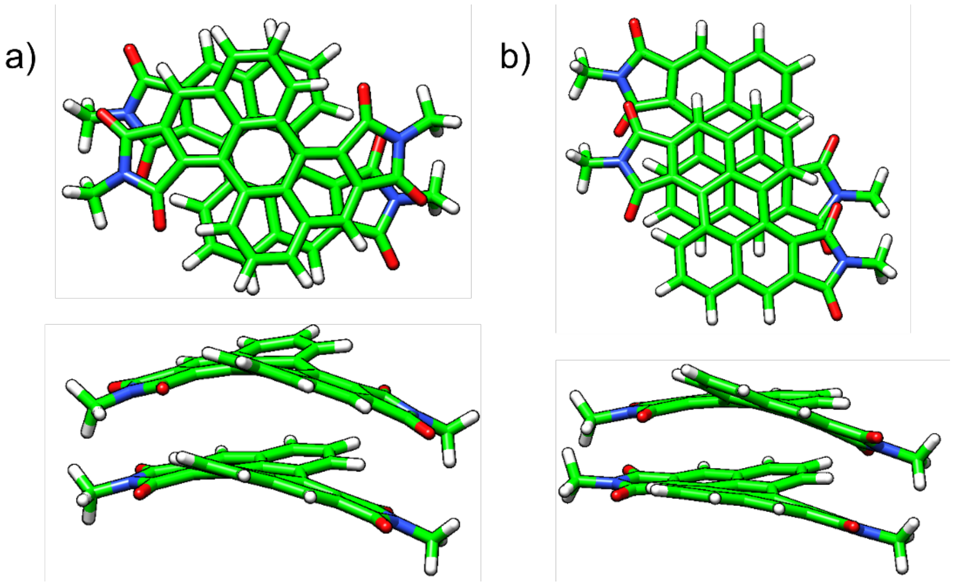

Figure S19. Simplified *D1b* (a) and *D2b* (b) models based on the previously-optimized *D1a* and *D2a* dimers (Figure S18) used for the TDDFT calculations at the ωB97X-D/cc-pVDZ level.

Based on the TDDFT calculations for dimers *D1b* and *D2b* and our own implementation of the powerful multistate and multifragment FPHD diabatization scheme,[S10] we have estimated the diabatic energies for the most relevant low-lying excited states of Frenkel and CT character and the excitonic/electronic couplings at the Franck–Condon region (Table S2). The charge-transfer singlet excited states are computed to be not degenerate in energy because the electronic structure calculations are performed in dimeric systems where the two molecules are not strictly equivalent. That energy offset between the charge transfer states is likely to decrease or be totally mitigated in a periodic supramolecular polymer. For both dimers (*D1b* and *D2b*), ωB97X-D predicts the CT states above the Frenkel-type states in the 0.30 - 0.42 eV range, being this

difference smaller for *D1b* (0.30 eV) compared to *D2b* (0.42 eV). Note that the energy characterization of the CT states is always a difficult task for TDDFT calculations and, consequently, our calculations can help us to set a plausible range for the Δ_{CT} parameter (0.20 - 0.50 eV range) in the FCTH Hamiltonian (Eq. S3). It should be stressed that Δ_{CT} is important for the shape of the simulated absorption spectra of the supramolecular aggregates $AggI_H$ and $AggII_n$ (Figure 5 and Figures S12-S14). In terms of excitonic/electronic couplings, there is a significant difference between *D1b* and *D2b*; while the short-range interactions (t_h and t_e) are the dominant ones for *D2b* compared to the long-range Coulombic interactions, this trend is less evident for *D1b* with similar values for both long-range and short-range interactions.

Table S2. Diabatic energies and excitonic/electronic couplings computed for the low-lying excited states of dimers *D1b* and *D2b* using the FPHD diabatization method and the *ω*B97X-D functional with the cc-pVDZ basis set.

	D1b	*D2b*
$E_{FE1} = E_{FE2}$ (eV)	2.98	2.95
E_{CT1} (eV)	3.28	3.37
E_{CT2} (eV)	3.59	3.66
J_{Coul} (eV)	0.070	0.057
t_h (eV)	–0.015	–0.141
t_e (eV)	–0.087	–0.124

Apart from the diabatic energies and excitonic/electronic couplings, it is necessary to set the parameter concerning the local electron-vibration coupling; in particular, the frequency of the effective vibration ($\hbar\omega_{vib}$) that modulates the diagonal energies and the HR factors (λ_0^2, λ_+^2 and λ_-^2). $\hbar\omega_{vib}$ and λ_0^2 have been extracted from the vibronic progression of the experimental absorption spectra for the monomeric ZPDI **1** ($\hbar\omega_{vib}$ = 1220 cm^{-1} and $\lambda_0^2 = 0.8$). For the ionic HR factors (λ_+^2 and λ_-^2), we have used the same value as λ_0^2. On the other hand, $E_{S_1} = \hbar\omega_{0-0}$ has been also taken from the first vibronic band ($A_{0\text{-}0}$) experimentally registered for the monomeric Z-PDI **1** (Figure 1). Table S3 summarizes all the parameters used for the simulation of the absorption spectra of monomer Z-PDI **1** and supramolecular aggregates $AggI_H$ and $AggII_n$ shown in Figure 5 of the main text.

Table S3. Set of parameters used for the simulation of the absorption spectra of Z-PDI **1** in its monomeric and polymeric forms ($AggI_H$ and $AggII_n$) shown in Figure 5 in the main text.

	monomer	$AggI_H$	$AggII_n$
n	1	15	15
$\hbar\omega_{0-0}$ (eV)	2.56	2.56	2.56
Δ_{CT_1} (eV)	-	0.30	0.42
Δ_{CT_2} (eV)		0.61	0.71
J_{Coul} (eV)	-	0.070	0.057
t_h (eV)	-	–0.015	–0.141
t_e (eV)	-	–0.087	–0.124
$\hbar\omega_{vib}$ (eV)	0.151	0.151	0.151
λ_0^2	0.8	0.8	0.8
$\lambda_+^2 = \lambda_-^2$	0.8	0.8	0.8

Table S4 collects all the parameters used for the simulation of the absorption spectra of monomer Z-PDI **1** and the supramolecular aggregate $AggII_n$ shown in Figures S12-S14, where the transfer integrals (t_h and t_e) and the energy position of the CT states (now degenerate in energy) have been systematically varied.

Table S4. Set of parameters used for the simulation of the absorption spectra of Z-PDI **1** in its monomeric and supramolecular aggregate $AggII_n$ shown in Figures S12-S14.

	monomer	*AggII*
n	1	15
$\hbar\omega_{0-0}$ (eV)	2.56	2.56
J_{Coul} (eV)	-	0.057
$\hbar\omega_{vib}$ (eV)	0.151	0.151
λ_0^2	0.8	0.8
$\lambda_+^2 = \lambda_-^2$	0.8	0.8

7. References

S1. Iseki, S.; Nonomura, K; Kishida, S.; Ogata, D.; Yuasa, J. Zinc-Ion-Stabilized Charge-Transfer Interactions Drive Self-Complementary or Complementary Molecular Recognition. *J. Am. Chem. Soc.* **2020**, *142*, 15842–15851.

S2. Rao, B. B.; Wei, J.-R.; Lin, C.-H. New Synthetic Routes to Z-Shape Functionalized Perylenes. *Org. Lett.* **2012**, *14*, 3640–3643.

S3. Greciano, E. E.; Sánchez, L. Seeded Supramolecular Polymerization in a Three-Domain Self-Assembly of an N-Annulated Perylenetetracarbox-amide. *Chem. Eur. J.* **2016**, *22*, 13724–13730.

S4. Korevaar, P. A.; Schaefer, C.; De Greef, T. F. A.; Meijer, E. W. Controlling Chemical Self-Assembly by Solvent-Dependent Dynamics. *J. Am. Chem. Soc.* **2012**, *134*, 13482–13491.

S5. a) Becke, A. D. A New Mixing of Hartree–Fock and Local Density-Functional Theories. *J. Chem. Phys.* **1993**, *98*, 1372–1377; b) Grimme, S.; Antony, J.; Ehrlich, S.; Krieg, H. A Consistent and Accurate Ab Initio Parametrization of Density Functional Dispersion Correction (DFT-D) for the 94 Elements H-Pu. *J. Chem. Phys.* **2010**, *132*, 154104.

S6. Chai, J.-D.; Head-Gordon, M. Long-Range Corrected Hybrid Density Functionals with Damped Atom-Atom Dispersion Corrections. *Phys. Chem. Chem. Phys.* **2008**, *10*, 6615–6620.

S7. Dunning, T. H. Gaussian Basis Sets for Use in Correlated Molecular Calculations. I. The Atoms Boron through Neon and Hydrogen. *J. Chem. Phys.* **1989**, *90*, 1007–1023.

S8. Aragó, J.; Sancho-García, J. C.; Ortí, E.; Beljonne, D. Ab Initio Modeling of Donor–Acceptor Interactions and Charge-Transfer Excitations in Molecular Complexes: The Case of Terthiophene–Tetracyanoquinodimethane. *J. Chem. Theory Comput.* **2011**, *7*, 2068–2077.

S9. Frisch, M. J.; Trucks, G. W.; Schlegel, H. B.; Scuseria, G. E.; Robb, M. A.; Cheeseman, J. R.; Scalmani, G.; Barone, V.; Petersson, G. A.; Nakatsuji, H.; Li, X.; Caricato, M.; Marenich, A. V; Bloino, J.; Janesko, B. G.; Gomperts, R.; Mennucci, B.; Hratchian, H. P.; Ortiz, J. V; Izmaylov, A. F.; Sonnenberg, J. L.; Williams-Young, D.; Ding, F.; Lipparini, F.; Egidi, F.; Goings, J.; Peng, B.; Petrone, A.; Henderson, T.; Ranasinghe, D.; Zakrzewski, V. G.; Gao, J.; Rega, N.; Zheng, G.; Liang, W.; Hada, M.; Ehara, M.; Toyota, K.; Fukuda, R.; Hasegawa, J.; Ishida, M.; Nakajima, T.; Honda, Y.; Kitao, O.; Nakai, H.; Vreven, T.; Throssell, K.; Montgomery Jr., J. A.; Peralta, J. E.; Ogliaro, F.; Bearpark, M. J.; Heyd, J. J.; Brothers, E. N.; Kudin, K. N.; Staroverov, V. N.; Keith, T. A.; Kobayashi, R.; Normand, J.; Raghavachari, K.; Rendell, A. P.; Burant, J. C.; Iyengar, S. S.; Tomasi, J.; Cossi, M.; Millam, J. M.; Klene, M.; Adamo, C.; Cammi, R.; Ochterski, J. W.; Martin, R. L.; Morokuma, K.; Farkas, O.; Foresman, J. B.; Fox, D. J. Gaussian16, Revision A.03. Gaussian Inc. Wallingford CT 2016.

S10. Wang, Y.-C.; Feng, S.; Liang, W.; Zhao, Y. Electronic Couplings for Photoinduced Charge Transfer and Excitation Energy Transfer Based on Fragment Particle–Hole Densities. *J. Phys. Chem. Lett.* **2021**, *12*, 1032–1039.
